# Impact of Vaping Prevention Advertisements on US Adolescents: A Randomized Clinical Trial

**DOI:** 10.1001/jamanetworkopen.2022.36370

**Published:** 2022-10-13

**Authors:** Seth M. Noar, Nisha C. Gottfredson, Talia Kieu, Jacob A. Rohde, Marissa G. Hall, Haijing Ma, Nicholas J. Fendinger, Noel T. Brewer

**Affiliations:** 1 Hussman School of Journalism and Media, University of North Carolina, Chapel Hill; 2 Lineberger Comprehensive Cancer Center, University of North Carolina, Chapel Hill; 3 Department of Health Behavior, Gillings School of Global Public Health, University of North Carolina, Chapel Hill; 4 Carolina Population Center, University of North Carolina, Chapel Hill

## Abstract

**Importance:**

Understanding whether prevention advertisements reduce susceptibility to vaping is important owing to concerning levels of adolescent vaping.

**Objective:**

To examine whether vaping prevention advertisements from the US Food and Drug Administration (FDA) national Real Cost campaign lead to lower susceptibility to vaping among adolescents.

**Design, Setting, and Participants:**

For this 3-group randomized clinical trial with parallel assignment, participants were US adolescents aged 13 to 17 years who were susceptible to vaping or current e-cigarette users, recruited from online panels. Adolescents were randomized to 1 of 2 Real Cost vaping prevention trial groups (health harms– or addiction-themed advertisements) or to a control group (investigator-created neutral videos about vaping). Adolescents completed 4 weekly online surveys at visits 1 to 4 over a 3-week period. Data were analyzed from December 1, 2021, to August 25, 2022.

**Interventions:**

Adolescents saw 3 randomly ordered 30-second video advertisements online at each of 3 weekly study visits (visits 1, 2, and 3).

**Main Outcomes and Measures:**

The primary trial outcome was susceptibility to vaping. Surveys also assessed susceptibility to smoking cigarettes to examine any spillover effects of vaping prevention advertisements on smoking outcomes. Both susceptibility measures had 3 items and ranged from 1 (indicating not susceptible) to 4 (indicating highly susceptible). The primary analyses compared Real Cost groups (combined) with the control group, while exploratory analyses compared the Real Cost groups with each other.

**Results:**

Participants were 1514 adolescents (1140 [75.3%] boys; mean [SD] age, 15.22 [1.18] years), including 504 randomized to the Real Cost health harms group, 506 randomized to the Real Cost addiction group, and 504 randomized to the control group. Adolescents in the Real Cost groups (combined) had lower susceptibility to vaping at visit 4 than those in the control group (*b* = −0.21; 95% CI, −0.32 to −0.10). The Real Cost groups did not differ from one another on susceptibility to vaping (visit 4: *b* = −0.05; 95% CI, −0.17 to 0.07). Adolescents in the Real Cost groups (combined) also had lower susceptibility to smoking cigarettes than those in the control group (*b* = −0.21; 95% CI, −0.32 to −0.10). For both vaping and smoking, Real Cost groups had less positive attitudes (vaping: *b* = −0.27; 95% CI, −0.40 to −0.14; smoking: *b* = −0.23; 95% CI, −0.39 to −0.08) compared with the control group.

**Conclusions and Relevance:**

These findings suggest that vaping prevention advertisements from the FDA Real Cost campaign led to lower adolescent susceptibility to vaping and had beneficial spillover effects on cigarette smoking outcomes. Tobacco prevention campaigns can help reduce youth tobacco use.

**Trial Registration:**

ClinicalTrials.gov Identifier: NCT04836455

## Key Points

**Question:**

Do vaping prevention advertisements from the US Food and Drug Administration Real Cost national campaign reduce susceptibility to vaping among youth?

**Findings:**

In this randomized clinical trial that included 1514 adolescents, Real Cost vaping prevention advertisements reduced susceptibility to vaping compared with control videos. Real Cost advertisements also reduced susceptibility to smoking cigarettes compared with control videos.

**Meaning:**

These findings suggest that vaping prevention advertisements reduced the extent to which youth were open to vaping and also had beneficial effects on cigarette smoking outcomes.

## Teaser

This randomized clinical trial examines whether vaping prevention advertisements from the Real Cost campaign lead to lower susceptibility to vaping among adolescents.

## Introduction

Adolescent vaping is a major public health concern. More than 2 million US middle and high school students used e-cigarettes as of 2021.^1^ While this rate of use is lower than the extraordinarily high level of youth vaping in 2019,^2^ use remains a concern. Vaping puts youth at risk of nicotine addiction^3,4^ and increases their chances of using other tobacco products.^5^ e-Cigarette aerosols also contain toxic chemicals^4^ which research suggests may cause lung diseases.^6,7^

Tobacco prevention campaigns are an evidence-based approach to reducing tobacco use among youth.^8,9,10^ Single-exposure experiments suggest vaping prevention messages may increase risk beliefs^11,12,13^ and reduce openness to vaping,^12,14^ but to our knowledge, researchers have yet to conduct randomized clinical trials (RCTs) with repeated exposure to messages over time. Moreover, a better understanding of how messages about nicotine addiction and health harms affect youth is needed, given the widespread use of these themes in vaping prevention campaigns.^15,16,17,18^

Vaping prevention advertisements could also have unintended consequences.^19^ Exposure to messages warning about the harms of vaping could inadvertently encourage use of other more harmful tobacco products, such as combustible cigarettes, if youth perceive vaping to be more harmful than smoking.^20,21,22^ Studies are needed to examine unintended effects, given the widespread exposure of young people to vaping prevention harms messages.^23^

In this study, we evaluated the impact of vaping prevention advertisements from the US Food and Drug Administration (FDA) Real Cost national vaping prevention campaign among a national convenience sample of US adolescents. We hypothesized that viewing Real Cost advertisements leads to lower susceptibility to vaping. We also explored the relative efficacy of health harms– vs addiction-themed advertisements and the potential impact of Real Cost advertisements on cigarette smoking outcomes.

## Methods

This RCT was approved by the University of North Carolina institutional review board. All parents or legal guardians of participants provided informed parental consent online, and all participants provided assent prior to participation. The trial protocol and statistical analysis plan are provided in Supplement 1. This study follows Consolidated Standards of Reporting Trials (CONSORT) reporting guideline for RCTs.

### Participants

We recruited adolescents living in the US from online panels administered by Qualtrics. Eligibility criteria were age 13 through 17 years, able to read and speak in English, able to take an online survey in English, and susceptible to e-cigarette use, as shown by an answer of 2 or greater on any of 5 e-cigarette susceptibility items.^24^ The susceptibility items assessed the extent to which adolescents were open to vaping, with a 4-point response scale that ranged from definitely not (coded as 1) to definitely yes (coded as 4).

**Figure 1.**
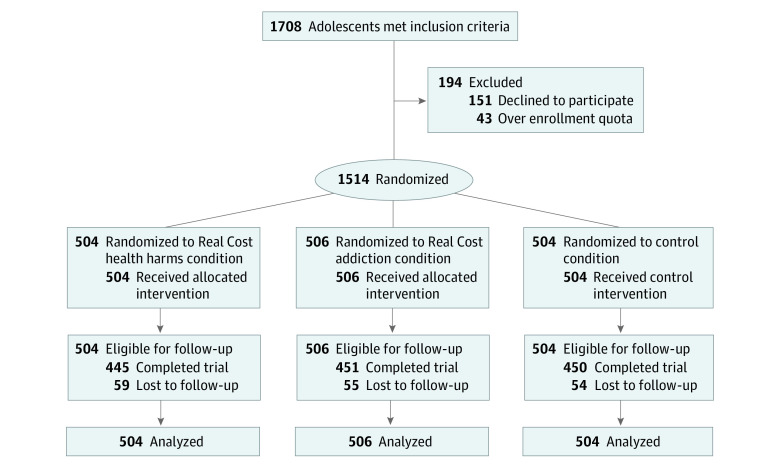
Participant Recruitment Flowchart

Recruitment took place from September to November 2021. Of 1708 adolescents who met inclusion criteria, 151 declined to participate and 43 were screened but unable to enroll because the study quota had been met (Figure 1). Demographic characteristics, including gender, racial, ethnic, and sexual orientation identities, were determined via self-report. Race was classified as Asian, Black, multiracial, Native Hawaiian or other Pacific Islander, or White. Participants were categorized as multiracial if they selected more than 1 race category. The other category was used for participants who selected the other category, but they were able to write in a racial identity if they wished, including American Indian or Alaska Native, Dominican, Fijian, and Mexican. Participants could also report Hispanic ethnicity. Race and ethnicity were included in analyses given historical disparities in tobacco use by race and ethnicity.

### Procedures

#### Trial Design and Protocol

We conducted a 3-group RCT with parallel assignment. Adolescents participated in 4 weekly online visits over a 3-week period, completing a survey at each visit. Exposures to campaign advertisements occurred at visits 1, 2, and 3. At the start of the trial (visit 1), participants were randomized to see a series of advertisements from 1 of 2 FDA Real Cost vaping prevention advertisement trial groups (focusing on health harms or addiction) or to a control group (investigator-created neutral videos about vaping). Participants were randomized without replacement to 1 of the 3 trial groups using the Qualtrics randomizer based on a single allocation ratio. In each group, participants viewed the same 3 advertisements per session, in a random order, corresponding to the trial group they were assigned. Participants were told they were going to view 3 short advertisements, but received no information on the content or number of groups. The research team was not blinded after assignment because interventions were administered online.

At each of the visits (visits 1-4), participants completed surveys reporting primary and secondary outcomes. Participants received incentives in the form of points for taking the surveys, with a total cash equivalent of $35 after completing the trial ($5 for the first and second surveys, $10 for the third survey, and $15 for the final survey). A pilot study with 51 participants was conducted prior to the trial to test trial procedures.

#### Intervention Messages

Participants in both intervention groups viewed three 30-second advertisements from the Real Cost campaign, corresponding to 1 of the 2 major themes of the campaign. Participants in the health harms intervention group viewed advertisements about toxic substances and lung damage, while participants in the addiction intervention group viewed advertisements about consequences of nicotine addiction. Participants in the control group viewed three 30-second investigator-created neutral videos about vaping consisting of black text on a white screen with a narrator reading the text. The content of these advertisements included product definitions, farming practices, and manufacturing practices adapted from Wikipedia^25^ and other sources (eTable 1 in Supplement 2).

### Measures

At the first session (visit 1), each participant reported vaping and smoking behaviors in the survey, then viewed advertisements that corresponded to their group assignment. They completed measures assessing message reactions after viewing each advertisement, after which they completed measures of susceptibility to vaping and smoking, vaping and smoking beliefs, and demographic characteristics. For the subsequent weeks of the trial (visits 2 and 3), participants completed surveys with measures of susceptibility to vaping and smoking, vaping and smoking beliefs, and vaping and smoking behavior. Then, they viewed the same 3 advertisements from their assigned group and completed the message reactions items. At visit 4, participants completed surveys assessing susceptibility to vaping and smoking, vaping and smoking beliefs, and vaping and smoking behavior (eFigure in Supplement 2).

#### Primary Outcome

Susceptibility to vaping at visit 4 was the primary trial outcome. Susceptibility is prospectively associated with vaping behavior, including when examined as a scaled score in which higher values are associated with both greater initiation and current use of vapes.^26,27^

We assessed susceptibility with a 3-item susceptibility to vaping scale, similar to other studies.^28,29,30,31^ This scale assesses the extent to which adolescents are open to vaping, with a 4-point response scale ranging from definitely not (coded as 1) to definitely yes (coded as 4) (eTable 2 and eTable 3 in Supplement 2). We calculated a susceptibility score by calculating the mean of the 3 items, with higher scores representing higher susceptibility (Cronbach α = .93). Susceptibility was measured after advertisement exposure at visit 1 to capture immediate advertisement impact and before advertisement exposure at visits 2 through 4 to capture advertisement impact over time.

#### Secondary Outcomes

The survey assessed several secondary outcomes hypothesized to elicit behavior change. Response scales for most secondary outcomes had 5 points, coded such that higher scores represented a greater amount of the construct. For multi-item scales, overall scale scores were the mean of the items.

##### Message Reactions

A single item assessed attention to the message and a 3-item scale assessed negative affect (α = .83).^32^ Message reactions were measured immediately following advertisement exposure. These measures were last assessed at visit 3 because the advertisements were not shown at visit 4.

##### Vaping Outcomes

Three-item scales assessed cognitive elaboration (α = .92),^33^ social interactions (α = .94),^34^ vaping health harm risk beliefs (α = .92), vaping addiction risk beliefs (α = .89),^35^ and vaping attitudes (α = .91).^36^ Participants reported the number of days they vaped over the past 7 days at each visit. These outcomes were measured after advertisement exposure at visit 1 and before advertisement exposure at visits 2 through 4, except for days vaped which was always measured before advertisement exposure.

##### Smoking Outcomes

Single items assessed smoking health harm risk belief, addiction risk belief, and smoking attitude. Susceptibility to smoking was assessed with a 3-item scale (α = .95). Participants reported the number of days they smoked over the past 7 days at each visit. We chose to retain the continuous measures of number of days vaping and smoking rather than dichotomizing these outcomes (as proposed in our trial protocol in Supplement 1) because we found substantial variability in number of days that these products were used.

### Statistical Analysis

Our target sample size was 1500, which accounted for up to 33% of participants dropping out during the trial. With an estimated intraclass correlation of 0.70, the trial had power to detect an effect size of *d* = 0.25 or larger between intervention groups (combined) and the control group.^12^

Analyses used an intent-to-treat approach that included all data collected from enrolled participants regardless of trial attrition, with full information maximum likelihood estimation to limit missing data assumptions.^37^ We used generalized linear mixed modeling software with random effects to account for individual differences to model the effect of trial group on the repeated primary and secondary outcomes.^38,39,40^ After establishing the unconditional growth model for the repeated outcomes, we regressed the random effects associated with the intercept and slope factors associated with visit number on a binary indicator of trial group (Real Cost [coded as 1] vs control [coded as 0]). The intercept was fixed at the last visit so main effects of trial group indicate a difference between groups at the last time point. We used a log link for Poisson-distributed count outcomes and the identity link for all other outcomes.

Next, for individuals randomized to the 2 Real Cost intervention groups, we repeated the same analyses to compare primary and secondary outcomes for the Real Cost health harms group (coded as 1) vs the Real Cost addiction group (coded as 0). We report all model findings as unstandardized regression coefficients (*b*) with 95% CIs. Additional analytic details are provided in eAppendix in Supplement 2.

We also explored whether the effect of Real Cost advertisement groups (vs control) on the primary trial outcome varied as a function of gender (1 = boys; 0 = girls), age (treated as nominal using dummy codes to avoid the linearity assumption), race (1 = White; 0 = other race, including individuals identifying as Asian, Black, multiracial, Native Hawaiian or other Pacific Islander, or another race), ethnicity (1 = Hispanic; 0 = not Hispanic), sexual orientation (1 = heterosexual; 0 = lesbian, gay, bisexual, or other orientation), parental education (treated as nominal using dummy codes), whether the adolescent lived with a tobacco user (1 = yes; 0 = no), and vaping status at trial enrollment (1 = vaped in past 30 days; 0 = had not vaped in past 30 days). For these analyses, we added the main effects of demographics and then interactions between demographics and the trial indicator to the model predicting vaping susceptibility from trial group. To avoid collinearity, we tested each demographic variable independently. We probed statistically significant interactions to examine the nature of moderation effects. We applied the Benjamini-Hochberg adjustment^41^ for the moderation analyses to retain an overall false discovery rate of 5% across multiple correlated outcomes.^42^

*P* values were 2-sided, and statistical significance was set at *P* < .05. Analyses were conducting using R Studio version 4.0.4 (R Project for Statistical Computing). Data were analyzed from December 1, 2021, to August 25, 2022.

## Results

A total of 1514 participants (1140 [75.3%] boys; mean [SD] age, 15.22 [1.18] years) completed the visit 1 survey, with 504 participants (33%) assigned to the Real Cost health harms group, 506 participants (33%) to the Real Cost addiction group, and 504 participants (33%) to the control group (Table 1; eTable 4 in Supplement 2). Retention at visit 4 was 91%. Beyond participants lost to follow up, the primary outcome had no missing data, and secondary outcomes had less than 1% missing data (eTable 5 in Supplement 2). Assigned group was not associated with missing data or attrition. Most participants were heterosexual (1432 participants [94.6%]) and currently in high school (1067 participants [70.6%]). There were 14 Asian participants (0.9%), 371 Black participants (24.5%), 33 participants identifying as multiple races (2.2%), 4 Native Hawaiian or other Pacific Islander participants (0.3%), and 1081 White participants (71.4%). There were 176 participants (11.6%) who identified as Hispanic ethnicity. As expected, past 30-day tobacco use was very common, with 993 participants (65.6%) reporting vaping, 895 participants (59.1%) reporting smoking cigarettes, and 950 participants (62.7%) reporting using other tobacco products.

**Table 1.** Participant Characteristics

Characteristic	Adolescents, No. (%)			
	Overall (N = 1514)	Real Cost		Control (n = 504)
		Health harms (n = 504)	Addiction (n = 506)	
Age, mean (SD), y	15.22 (1.18)	15.21 (1.17)	15.18 (1.19)	15.28 (1.18)
Gender identity				
Boys	1140 (75.3)	372 (73.8)	375 (74.1)	393 (78.0)
Girls	358 (23.6)	126 (25.0)	122 (24.1)	110 (21.8)
Other responses	15 (1.1)	6 (1.2)	9 (1.8)	1 (0.2)
Race				
Asian	14 (0.9)	5 (1.0)	3 (0.6)	6 (1.2)
Black	371 (24.5)	137 (27.2)	116 (22.9)	118 (23.4)
Multiracial	33 (2.2)	12 (2.4)	13 (2.6)	8 (1.6)
Native Hawaiian or other Pacific Islander	4 (0.3)	2 (0.4)	2 (0.4)	0 (0.0)
White	1081 (71.4)	345 (68.5)	367 (72.5)	369 (73.2)
Other^a^	10 (<0.1)	3 (<0.1)	4 (<0.1)	3 (<0.1)
Missing	1 (<0.1)	0 (0.0)	1 (<0.1)	0 (0.0)
Hispanic ethnicity	176 (11.6)	51 (10.1)	64 (12.6)	61 (12.1)
Sexual orientation				
Heterosexual	1432 (94.6)	476 (94.4)	471 (93.1)	485 (96.2)
Lesbian, gay, bisexual, pansexual, or queer	61 (4.1)	21 (4.2)	27 (5.3)	13 (2.6)
Prefer not to say or missing	20 (1.3)	7 (1.4)	8 (1.6)	6 (1.2)
Own education				
<High school	233 (15.4)	77 (15.3)	80 (15.8)	76 (15.1)
Some high school	1067 (70.6)	360 (71.4)	355 (70.2)	352 (69.8)
High school or GED	123 (8.1)	38 (7.5)	41 (8.1)	44 (8.7)
Some college	85 (5.6)	26 (5.2)	29 (5.7)	30 (6.0)
Dropped out of school	5 (0.3)	3 (0.6)	0 (0.0)	2 (0.4)
Missing	1 (0.1)	0 (0.0)	1 (0.2)	0 (0.0)
Mother’s education				
<Bachelor’s degree	262 (17.3)	85 (16.9)	102 (20.1)	75 (14.9)
Bachelor’s degree	533 (35.2)	178 (35.3)	174 (34.4)	181 (35.9)
Master’s degree	578 (38.2)	194 (38.5)	184 (36.4)	200 (39.7)
Doctorate degree	123 (8.1)	41 (8.1)	41 (8.1)	41 (8.1)
Missing	18 (1.2)	6 (1.2)	5 (1.0)	7 (1.4)
Father’s education				
<Bachelor’s degree	169 (11.1)	55 (11)	67 (13.3)	47 (8.8)
Bachelor’s degree	353 (23.3)	127 (25.2)	108 (21.3)	118 (23.4)
Master’s degree	699 (46.2)	234 (46.4)	229 (45.3)	236 (47.4)
Doctorate degree	273 (18.0)	82 (16.3)	94 (18.6)	97 (19.2)
Missing	20 (1.3)	6 (1.2)	8 (1.6)	6 (1.2)
Lived with somebody who				
Smoked cigarettes	539 (35.6)	161 (31.9)	179 (35.4)	199 (39.5)
Used e-cigarettes or vapes	443 (29.3)	135 (26.8)	144 (28.5)	164 (32.5)
Used chewing tobacco, snuff, or dip	218 (14.4)	72 (14.3)	73 (14.4)	73 (14.5)
Smoked cigars, cigarillos, or little cigars	203 (13.4)	69 (13.7)	57 (11.3)	77 (15.3)
Used another form of tobacco	119 (7.9)	43 (8.5)	39 (7.7)	37 (7.3)
Tobacco use, in the past 30 d				
Used e-cigarette	993 (65.6)	324 (64.3)	336 (66.4)	333 (66.1)
Used cigarette	895 (59.1)	292 (57.9)	297 (58.7)	306 (60.7)
Used OTP	950 (62.7)	320 (63.5)	305 (60.3)	325 (64.5)

Abbreviations: GED, general educational development; OTP, other tobacco product.

^a^ Includes participants who self-reported another race not listed in the table, including American Indian or Alaska Native, Dominican, Fijian, and Mexican.

### Real Cost vs Control

#### Susceptibility to Vaping

**Figure 2.**
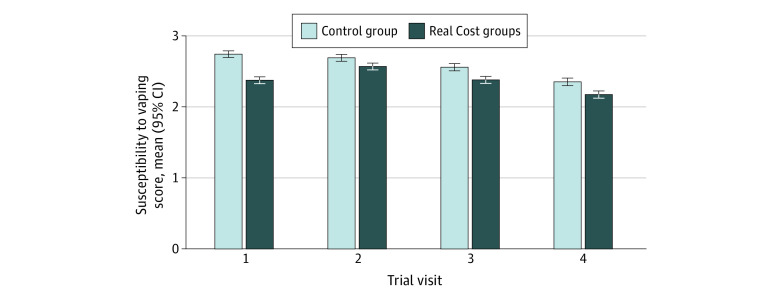
Effects of Real Cost Interventions (Combined) on Susceptibility to Vaping Error bars indicate 95% CIs.

The Real Cost groups had lower vaping susceptibility at visit 4 than the control group (*b* = −0.21; 95% CI, −0.32 to −0.10), the primary trial outcome. The effect of trial group on susceptibility was largest at visit 1 (*b* = −0.37; 95% CI, −0.47 to −0.26), smallest at visit 2 (*b* = −0.15; 95% CI, −0.26 to −0.04), and larger again at visit 3 (*b* = −0.20; 95% CI, −0.31 to −0.09) and visit 4 (Figure 2). The effect on susceptibility to vaping at visit 4 did not differ in size across any demographic group except for race. The Real Cost advertisements were more effective for adolescents who identified as a race other than White than for adolescents who identified as White (simple slopes: non-White adolescents: *b* = −0.39; 95% CI, −0.64 to −0.14; White adolescents: *b* = −0.08; 95% CI, −0.22 to 0.06; interaction *P* < .001).

#### Message Reactions

Participants in the Real Cost groups reported higher attention than participants in the control group at all visits (*b* = 0.43 to 0.44). Participants in Real Cost groups also reported more negative affect than participants in the control group at all visits (*b* = 0.59 to 0.67) (Table 2).

**Table 2.** Impact of Real Cost Advertisements vs Control Videos on Trial Outcomes^a^

Measure	Vaping outcomes				Smoking outcomes			
	Mean (SD)		Difference between groups		Mean (SD)		Difference between groups	
	Both Real Cost groups	Control group	*b* (95% CI)	*P* value	Both Real Cost groups	Control group	*b* (95% CI)	*P* value
**Susceptibility**								
Visit 1	2.38 (0.96)	2.75 (0.94)	−0.37 (−0.47 to −0.26)	<.001	2.27 (1.02)	2.48 (1.00)	−0.21 (−0.33 to −0.11)	<.001
Visit 2	2.58 (0.96)	2.70 (0.97)	−0.15 (−0.26 to −0.04)	.008	2.37 (1.03)	2.46 (1.01)	−0.13 (−0.24 to −0.01)	.03
Visit 3	2.39 (0.99)	2.57 (1.02)	−0.20 (−0.31 to −0.09)	<.001	2.19 (1.02)	2.33 (1.03)	−0.17 (−0.28 to −0.05)	.005
Visit 4	2.18 (1.03)	2.36 (1.07)	−0.21 (−0.32 to −0.10)	<.001	2.04 (1.02)	2.21 (1.05)	−0.21 (−0.32 to −0.10)	<.001
**Attention**								
Visit 1	4.36 (0.85)	3.91 (1.18)	0.44 (0.34 to 0.54)	<.001	NA	NA	NA	NA
Visit 2	4.44 (0.83)	4.03 (1.11)	0.44 (0.33 to 0.54)	<.001	NA	NA	NA	NA
Visit 3	4.49 (0.78)	4.10 (1.11)	0.43 (0.33 to 0.54)	<.001	NA	NA	NA	NA
**Negative affect**								
Visit 1	3.14 (1.09)	2.47 (1.21)	0.67 (0.54 to 0.80)	<.001	NA	NA	NA	NA
Visit 2	3.22 (1.16)	2.62 (1.31)	0.59 (0.46 to 0.72)	<.001	NA	NA	NA	NA
Visit 3	3.31 (1.16)	2.68 (1.33)	0.63 (0.50 to 0.76)	<.001	NA	NA	NA	NA
**Cognitive elaboration**								
Visit 1	3.09 (1.24)	2.90 (1.25)	0.20 (0.07 to 0.33)	.002	NA	NA	NA	NA
Visit 2	3.27 (1.17)	3.20 (1.23)	0.07 (−0.07 to 0.20)	.34	NA	NA	NA	NA
Visit 3	3.47 (1.14)	3.35 (1.18)	0.10 (−0.03 to 0.24)	.08	NA	NA	NA	NA
Visit 4	3.58 (1.20)	3.43 (1.28)	0.13 (−0.01 to 0.26)	.06	NA	NA	NA	NA
**Social interactions**								
Visit 1	2.74 (1.38)	2.65 (1.36)	0.09 (−0.06 to 0.23)	.25	NA	NA	NA	NA
Visit 2	2.96 (1.34)	2.97 (1.35)	−0.01 (−0.16 to 0.14)	.86	NA	NA	NA	NA
Visit 3	3.17 (1.30)	3.03 (1.36)	0.13 (−0.02 to 0.29)	.08	NA	NA	NA	NA
Visit 4	3.27 (1.38)	3.19 (1.45)	0.08 (−0.07 to 0.23)	.28	NA	NA	NA	NA
**Health harm risk beliefs**								
Visit 1	4.02 (1.00)	3.54 (1.19)	0.48 (0.36 to 0.60)	<.001	3.94 (1.21)	3.66 (1.32)	0.28 (0.14 to 0.41)	<.001
Visit 2	3.84 (1.08)	3.69 (1.18)	0.16 (0.04 to 0.28)	.01	3.80 (1.27)	3.72 (1.28)	0.09 (−0.05 to 0.23)	.21
Visit 3	3.99 (1.08)	3.80 (1.17)	0.19 (0.07 to 0.31)	.002	3.87 (1.28)	3.78 (1.27)	0.07 (−0.07 to 0.22)	.31
Visit 4	4.09 (1.05)	3.85 (1.18)	0.25 (0.13 to 0.37)	<.001	4.00 (1.25)	3.83 (1.29)	0.17 (0.03 to 0.31)	.01
**Addiction risk beliefs**								
Visit 1	3.70 (1.15)	3.41 (1.15)	0.28 (0.16 to 0.41)	<.001	3.77 (1.27)	3.53 (1.38)	0.23 (0.09 to 0.37)	.001
Visit 2	3.63 (1.13)	3.55 (1.16)	0.09 (−0.04 to 0.22)	.19	3.67 (1.31)	3.63 (1.36)	0.03 (−0.12 to 0.18)	.68
Visit 3	3.68 (1.15)	3.52 (1.22)	0.15 (0.02 to 0.28)	.02	3.75 (1.31)	3.68 (1.30)	0.05 (−0.10 to 0.20)	.53
Visit 4	3.78 (1.15)	3.58 (1.28)	0.21 (0.08 to 0.34)	.006	3.81 (1.33)	3.76 (1.37)	0.05 (−0.10 to 0.20)	.50
**Attitudes**								
Visit 1	2.54 (1.16)	3.00 (1.17)	−0.46 (−0.59 to −0.33)	<.001	2.35 (1.38)	2.56 (1.35)	−0.20 (−0.35 to −0.05)	.01
Visit 2	2.80 (1.21)	2.99 (1.23)	−0.21 (−0.35 to −0.08)	.002	2.53 (1.46)	2.58 (1.44)	−0.10 (−0.25 to 0.06)	.22
Visit 3	2.57 (1.27)	2.78 (1.29)	−0.22 (−0.36 to −0.09)	.001	2.33 (1.43)	2.52 (1.45)	−0.22 (−0.38 to −0.06)	.01
Visit 4	2.36 (1.27)	2.60 (1.33)	−0.27 (−0.40 to −0.14)	<.001	2.14 (1.40)	2.35 (1.46)	−0.23 (−0.39 to −0.08)	.003
**Days used in the past week**								
Visit 1	2.92 (1.81)	2.95 (1.88)	0.93 (0.78 to 1.10)	.40	2.86 (1.85)	2.78 (1.80)	0.90 (0.74 to 1.10)	.30
Visit 2	2.32 (2.15)	2.38 (2.18)	0.89 (0.74 to 1.05)	.27	1.97 (2.03)	2.01 (2.03)	0.86 (0.71 to 1.05)	.14
Visit 3	2.18 (2.19)	2.31 (2.15)	0.86 (0.72 to 1.03)	.09	1.82 (1.99)	1.90 (1.99)	0.84 (0.69 to 1.03)	.51
Visit 4	1.92 (2.14)	2.14 (2.11)	0.82 (0.69 to 0.98)	.03	1.62 (1.97)	1.80 (1.96)	0.80 (0.65 to 0.97)	.03

Abbreviation: NA, not appliable.

^a^ Results are from linear mixed models, including unstandardized regression coefficients (*b*). Because days used in the past week had a Poisson-distributed count outcome, analyses used a log link function; these coefficients have been exponentiated and reflect multiplicative effects of trial group.

#### Vaping Outcomes

Participants in the Real Cost groups reported higher cognitive elaboration than those in the control group, but only at visit 1 (*b* = 0.20; 95% CI, 0.07 to 0.33). There was no effect on social interactions. Participants in the Real Cost groups had higher addiction risk beliefs at visits 1, 3, and 4 (*b* = 0.15 to 0.28) compared with the control group. The Real Cost groups also had higher health harm risk beliefs (*b* = 0.16 to 0.48) and more negative vaping attitudes (*b* = −0.21 to −0.46) at all visits (Table 2). Finally, participants in the Real Cost groups had fewer days vaped per week at visit 4 compared with the control group (*b* = 0.82; 95% CI, 0.69 to 0.98).

#### Smoking Outcomes

Participants in the Real Cost groups were less susceptible to smoking cigarettes than participants in the control group at all visits (*b* = −0.13 to −0.21) (Table 2). Participants in the Real Cost groups also had higher smoking addiction risk beliefs at visit 1 (*b* = 0.23; 95% CI, 0.09 to 0.37) than those in the control group. The Real Cost participants had higher smoking health harm risk beliefs at visits 1 (*b* = 0.28; 95% CI, 0.14 to 0.41) and 4 (*b* = 0.17; 95% CI, 0.03 to 0.31) compared with control participants. Participants in the Real Cost groups had more negative smoking attitudes at visits 1, 3, and 4 (*b* = −0.20 to −0.23) and fewer days smoking cigarettes per week at visit 4 compared with control participants (*b* = 0.80; 95% CI, 0.65 to 0.97) (Table 2).

### Health Harms vs Addiction

Participants in the Real Cost health harms group had lower levels of susceptibility to vaping at visit 1 than those in the addiction group (*b* = −0.14; 95% CI, −0.27 to −0.01), but there were no differences at subsequent visits (Table 3). Participants in the Real Cost health harms group had higher attention at visits 1 (*b* = 0.11; 95% CI, 0.01 to 0.21) and 2 (*b* = 0.13; 95% CI, 0.02 to 0.24) and negative affect at all visits (*b* = 0.23 to 0.26) than those in the addiction group (Table 3). There were no differences between Real Cost groups on cognitive elaboration, social interactions, vaping addiction risk beliefs, vaping attitudes, or days vaped in the past week. Participants in the Real Cost health harms group had higher health harm risk beliefs than those in the addiction group at visit 1 (*b* = 0.18; 95% CI, 0.05 to 0.31), but there were no differences at subsequent visits. The Real Cost groups did not differ on any of the smoking outcomes (Table 3).

**Table 3.** Impact of Health Harms vs Addiction Real Cost Advertisements on Trial Outcomes^a^

Measure	Vaping outcomes				Smoking outcomes			
	Real Cost, mean (SD)		Difference between groups		Real Cost, mean (SD)		Difference between groups	
	Health harms	Addiction	*b* (95% CI)	*P* value	Health harms	Addiction	*b* (95% CI)	*P* value
**Susceptibility**								
Visit 1	2.31 (0.98)	2.45 (0.94)	−0.14 (−0.27 to −0.01)	.03	2.22 (1.00)	2.31 (1.03)	−0.08 (−0.20 to 0.05)	.20
Visit 2	2.53 (0.96)	2.62 (0.96)	−0.09 (−0.21 to 0.03)	.14	2.34 (1.03)	2.40 (1.03)	−0.08 (−0.21 to 0.05)	.24
Visit 3	2.37 (0.99)	2.40 (0.99)	−0.04 (−0.16 to 0.08)	.52	2.18 (1.03)	2.20 (1.01)	−0.03 (−0.16 to 0.10)	.63
Visit 4	2.16 (1.04)	2.21 (1.01)	−0.05 (−0.17 to 0.07)	.40	2.02 (1.02)	2.05 (1.02)	−0.04 (−0.17 to 0.09)	.57
**Attention**								
Visit 1	4.41 (0.84)	4.30 (0.87)	0.11 (0.01 to 0.21)	.04	NA	NA	NA	NA
Visit 2	4.49 (0.81)	4.38 (0.85)	0.13 (0.02 to 0.24)	.02	NA	NA	NA	NA
Visit 3	4.49 (0.79)	4.49 (0.77)	0.03 (−0.07 to 0.13)	.62	NA	NA	NA	NA
**Negative affect**								
Visit 1	3.25 (1.07)	3.03 (1.10)	0.23 (0.09 to 0.37)	.001	NA	NA	NA	NA
Visit 2	3.35 (1.13)	3.10 (1.17)	0.26 (0.11 to 0.41)	<.001	NA	NA	NA	NA
Visit 3	3.42 (1.13)	3.19 (1.19)	0.23 (0.08 to 0.38)	.003	NA	NA	NA	NA
**Cognitive elaboration**								
Visit 1	3.13 (1.26)	3.06 (1.23)	0.07 (−0.08 to 0.22)	.35	NA	NA	NA	NA
Visit 2	3.28 (1.15)	3.27 (1.19)	0.01 (−0.15 to 0.17)	.86	NA	NA	NA	NA
Visit 3	3.49 (1.16)	3.44 (1.13)	0.06 (−0.10 to 0.22)	.44	NA	NA	NA	NA
Visit 4	3.59 (1.20)	3.57 (1.21)	0.01 (−0.15 to 0.17)	.85	NA	NA	NA	NA
**Social interactions**								
Visit 1	2.81 (1.37)	2.66 (1.38)	0.14 (−0.03 to 0.31)	.10	NA	NA	NA	NA
Visit 2	2.97 (1.32)	2.95 (1.37)	0.05 (−0.12 to 0.22)	.60	NA	NA	NA	NA
Visit 3	3.19 (1.31)	3.16 (1.28)	0.05 (−0.12 to 0.22)	.58	NA	NA	NA	NA
Visit 4	3.33 (1.39)	3.20 (1.36)	0.13 (−0.04 to 0.30)	.15	NA	NA	NA	NA
**Health harm risk beliefs**								
Visit 1	4.11 (0.97)	3.93 (1.02)	0.18 (0.05 to 0.31)	.01	3.92 (1.23)	3.95 (1.19)	−0.02 (−0.17 to 0.14)	.76
Visit 2	3.83 (1.06)	3.84 (1.10)	−0.02 (−0.15 to 0.11)	.74	3.80 (1.27)	3.81 (1.27)	−0.01 (−0.17 to 0.15)	.92
Visit 3	3.98 (1.08)	4.00 (1.07)	−0.02 (−0.15 to 0.11)	.76	3.87 (1.26)	3.87 (1.30)	.01 (−0.15 to 0.18)	.95
Visit 4	4.09 (1.08)	4.09 (1.03)	−0.01 (−0.15 to 0.13)	.97	4.02 (1.22)	3.98 (1.28)	0.05 (−0.11 to 0.21)	.54
**Addiction risk beliefs**								
Visit 1	3.66 (1.20)	3.73 (1.09)	−0.07 (−0.22 to 0.08)	.37	3.72 (1.32)	3.81 (1.22)	−0.09 (−0.25 to 0.07)	.28
Visit 2	3.66 (1.11)	3.61 (1.15)	0.05 (−0.10 to 0.20)	.51	3.63 (1.34)	3.70 (1.29)	−0.07 (−0.24 to 0.10)	.40
Visit 3	3.69 (1.13)	3.68 (1.17)	0.01 (−0.14 to 0.16)	.88	3.70 (1.36)	3.79 (1.27)	−0.10 (−0.27 to 0.07)	.23
Visit 4	3.77 (1.18)	3.78 (1.13)	−0.02 (−0.17 to 0.13)	.82	3.79 (1.33)	3.82 (1.33)	−0.04 (−0.21 to 0.13)	.61
**Attitudes**								
Visit 1	2.48 (1.16)	2.60 (1.16)	−0.12 (−0.27 to 0.03)	.11	2.36 (1.38)	2.35 (1.37)	0.01 (−0.17 to 0.18)	.90
Visit 2	2.76 (1.20)	2.84 (1.22)	−0.09 (−0.24 to 0.06)	.24	2.52 (1.45)	2.54 (1.47)	−0.04 (−0.22 to 0.14)	.68
Visit 3	2.57 (1.27)	2.58 (1.27)	−0.03 (−0.19 to 0.13)	.73	2.35 (1.44)	2.32 (1.42)	0.02 (−0.16 to 0.20)	.85
Visit 4	2.35 (1.29)	2.37 (1.25)	−0.03 (−0.19 to 0.13)	.73	2.13 (1.38)	2.16 (1.42)	−0.04 (−0.22 to 0.14)	.68
**Days used in the past week**								
Visit 1	2.91 (1.82)	2.93 (1.81)	0.96 (0.78 to 1.19)	.67	2.81 (1.91)	2.90 (1.79)	0.94 (0.74 to 1.20)	.66
Visit 2	2.30 (2.15)	2.33 (2.14)	0.96 (0.78 to 1.18)	.70	1.94 (2.05)	2.00 (2.03)	0.93 (0.73 to 1.19)	.60
Visit 3	2.12 (2.16)	2.23 (2.21)	0.93 (0.76 to 1.15)	.51	1.77 (1.97)	1.86 (2.01)	0.93 (0.73 to 1.18)	.55
Visit 4	1.86 (2.08)	1.98 (2.19)	0.91 (0.74 to 1.12)	.37	1.61 (1.94)	1.63 (2.00)	0.95 (0.75 to 1.21)	.70

Abbreviation: NA, not applicable.

^a^ Results are from linear mixed models, including unstandardized regression coefficients (*b*). Because days used in the past week had a Poisson-distributed count outcome, analyses used a log link function; these coefficients have been exponentiated and reflect multiplicative effects of trial group.

## Discussion

Our RCT with a large sample of US adolescents found that vaping prevention advertisements from the FDA Real Cost campaign led to lower susceptibility to vaping, greater health harm and addiction risk beliefs about vaping, and less positive attitudes about vaping. We also found beneficial effects of vaping prevention advertisements on cigarette smoking outcomes, including lower susceptibility to smoking and less positive attitudes toward smoking. These latter findings are consistent with a recent study showing positive effects of youth tobacco prevention advertisements on nontargeted tobacco products,^43^ and a study of adult tobacco users that found e-cigarette warnings to have beneficial effects on cigarette smoking outcomes.^44^

The results of our trial extend those of single-exposure experiments^11,12^ and suggest that vaping prevention advertisements can have meaningful effects on youth’s beliefs about vaping. While the Real Cost vaping prevention campaign has primarily been disseminated through targeted digital media, studies indicate that the campaign has had wide reach.^23,45^ Thus, the campaign has likely discouraged youth from vaping and perhaps has helped mitigate the increases in youth vaping over the past several years.^2^ In doing so, it appears likely that the campaign has also discouraged youth from smoking cigarettes.

Our findings also provide important insights into message themes for vaping prevention campaigns. The health harms advertisements performed better than addiction advertisements on only a few outcomes and time points, and we interpret these differences as indicating only a marginal advantage for health harms advertisements. Prior research has found health harms package warnings to be more effective than addiction warnings for discouraging vaping,^44,46^ including among youth,^47^ raising the question of what accounts for our findings. One explanation may be that package warnings tend to focus only on the presence of nicotine itself. In contrast, video advertisements can visually demonstrate the consequences of nicotine addiction, something youth tend to discount.^48,49^ In our trial, the consequences featured in the addiction advertisements included negative effects on mood and loss of autonomy, which may be particularly salient to youth.^48^ It may be that by emphasizing the consequences of nicotine addiction that these advertisements performed similarly to health harm advertisements.^18^

The trial also revealed that Real Cost advertisements had similar effects across demographic groups. The exception was that advertisements had a greater impact on adolescents who identified as a race other than White vs whose who identified as White. While non-Hispanic White youth are most likely to report current e-cigarette use,^50^ non-Hispanic Black and Hispanic youth are more likely to start vaping earlier than non-Hispanic White youth,^51^ and communication campaigns may be capable of helping reduce this disparity. We had little representation among lesbian, gay, bisexual, pansexual, queer, or transgender youth, and future studies should examine the impact of vaping prevention advertisements among these populations. Future research should also examine what features make vaping prevention video advertisements effective and whether promising advertising features vary by sociodemographic and tobacco characteristics.^17^ Strengths of our trial include longitudinal exposure to messages, use of high-quality stimuli from a national campaign, recruitment of a large, at-risk sample, and high retention.

### Limitations

This study has some limitations, including structured exposure to vaping prevention advertisements (which differs from real-world exposure), the short 3-week study period, and self-reported tobacco use. Longer-term and observational studies evaluating the outcomes of the Real Cost campaign will further add to the evidence base about the effectiveness of vaping prevention campaigns for youth.

## Conclusions

Our randomized clinical trial found that vaping prevention advertisements focused on health harms and nicotine addiction led to lower susceptibility to vaping among youth in the US, while also discouraging cigarette smoking. These results should bolster efforts by the FDA and other public health authorities to communicate with youth about the adverse effects of vaping. Communication campaigns play a crucial role—alongside policy changes—in reducing youth vaping and its corresponding negative consequences.

## Notes

Supplement 1.
Trial Protocol and Statistical Analysis Plan

Supplement 2. **eTable 1.** Vaping Prevention Video Advertisements Used in the Trial **eFigure.** Trial Design **eTable 2.** Primary and Secondary Outcome Measures Used in the Trial **eTable 3.** RCT Survey Codebook for Visits 1-4
eReferences.
**eAppendix.** Additional Information on Statistical Models **eTable 4.** Participant Characteristics With Real Cost Groups Combined **eTable 5.** Item Completion at Visits 2, 3, and 4

Supplement 3.
Data Sharing Statement
