# Supplementary material for: Impact of Vaping Prevention Advertisements on US Adolescents: A Randomized Clinical Trial
Source: JAMA Netw Open. 2022 Oct 13;5(10):e2236370. doi: 10.1001/jamanetworkopen.2022.36370 (PMC9561946; doi:10.1001/jamanetworkopen.2022.36370)
Supplement: Supplement 1. — Trial Protocol and Statistical Analysis Plan [file jamanetwopen-e2236370-s001.pdf]

Sponsor: University of North Carolina, Chapel Hill
Collaborator: National Cancer Institute (NCI), Food and Drug Administration (FDA)
Information provided by (Responsible Party): University of North Carolina, Chapel Hill
ClinicalTrials.gov Identifier: NCT04836455

Brief Title Impact of Vaping Prevention Advertisements

Official Title: Impact of *The Real Cost* Vaping Prevention Advertisements on Adolescents

Brief Summary The purpose of this randomized controlled trial is to determine whether exposure to vaping prevention advertisements (ads) reduce susceptibility to vaping among adolescents. Previous studies have been informative, but they have tended to be one-time experimental studies that do not replicate the repeated exposures to ads that people have in the real world. This study addresses this issue by repeatedly exposing participants to vaping prevention ads over time. Participants will be adolescents aged 13-17 who currently vape or who are susceptible to vaping.

Participants will be randomly assigned to ad stimuli. They will be assigned to one of two *The Real Cost* trial arms—health harms or addiction—or to a control trial arm (probability of assignment is 1/3 for all trial arms). Participants in the study will take 4 online surveys over a 3-week period, once per week (At week 0, 1, 2, and 3). All participants will view randomized ad stimuli based on their trial arm and answer surveys items at each session.

Detailed description Tobacco prevention mass media campaigns are a key tool for reducing tobacco use among adolescents. While vaping has increased greatly among adolescents, there has been a dearth of research on effective communication strategies to reduce vaping. This study will test the impact of advertisements from the Food and Drug Administration's *The Real* Cost campaign on reducing susceptibility to vaping in a randomized controlled trial, illuminating whether such ads are effective as well as what themes are most effective (i.e., health harms, addiction). The investigators focus on adolescents (ages 13-17) who currently vape or are at risk of vaping.

Setting: The trial will be a longitudinal online study with 4 surveys over a 3 week period – 1 survey per week.

Recruitment: Adolescent participants will be recruited through Qualtrics, an online survey panel platform. Interested prospective participants will complete a screening questionnaire to determine their eligibility. If eligible, Qualtrics will invite them to enroll in the trial.

Informed Consent: Qualtrics will obtain parental consent online for adolescents who are eligible and interested in participating. After parental consent, adolescents will provide online assent prior to taking the survey.

Randomization: After providing informed assent, Qualtrics survey software will randomly assign participants to one of the three trial arms. Participants will have an equal chance of being randomized to any of the 3 trial arms.

Assessment: Participants in the study will take 4 surveys over a 3-week period. This will allow for multiple exposures to campaign ads. At week 0, we will randomize participants to 1 of 2 FDA *The Real Cost* vaping prevention ad trial arms (health harms or addiction) or to a control arm (investigator created neutral ads about vaping). In each condition, participants will view 3 ads at each session, in a random order, corresponding to the theme they were assigned. At the first session (week 0), each participant will complete measures of vaping and smoking behavior, and will then view the ads that correspond to their trial arm. They will complete measures assessing message reactions after viewing each ad, followed by measures of susceptibility to vaping, vaping and smoking beliefs, and finally questions assessing demographics. For week 1 and 2 assessments, participants will complete measures of susceptibility to vaping, vaping and smoking beliefs, and vaping and smoking behavior. They will then view the same 3 ads from their trial arm and complete the message reactions measures. For the week 3 assessment, participants will only complete measures of susceptibility to vaping, vaping and smoking beliefs, and vaping and smoking behavior. Each survey will take approximately 15 minutes.

Study Type: Interventional

Study Phase: n/a

Study Design: Allocation: Randomized
Endpoint Classification: Efficacy Study
Intervention Model: Parallel Assignment
Masking: Open Label
Primary Purpose: Prevention

Condition: Vaping behavior

Intervention

- Experimental: The FDA's *The Real Cost* vaping prevention ads – Health harms theme

  Three vaping prevention video ads from the FDA's *The Real Cost* prevention campaign about the health harms of vaping will be shown to participants at weeks 0, 1, and 2. Ads will be shown in a random order.

- Experimental: The FDA's *The Real Cost* vaping prevention ads – Addiction theme

  Three vaping prevention video ads from the FDA's *The Real Cost* prevention campaign about vaping addiction will be shown to participants at week 0, 1, and 2. Ads will be shown in a random order.

- Other: Neutral vaping ads

  Three neutral (i.e., purely informational and without graphics) vaping video ads developed by the Investigators will be shown to participants at weeks 0, 1, and 2. Ads will be shown in a random order.

Study Arm (s)

- Experimental: *The Real Cost* vaping prevention ads – Health effects theme
- Experimental: *The Real Cost* vaping prevention ads – Addiction theme

- Other: Neutral vaping ads

Publications n/a

Recruitment Information

Recruitment Status ICMJE: Not active

Actual Enrollment ICMJE: 1,514

Actual Completion Date: November 3, 2021

Actual Primary Completion Date: November 3, 2021

Eligibility Criteria ICMJE:

Inclusion Criteria:

- Between ages 13-17 years old (inclusive)
- Be able to read and speak English
- Be able to take an online survey in English
- Be susceptible to vaping

Exclusion Criteria:

- None

Gender: Both

Ages: 13-17

Accepts Healthy Volunteers: Yes

Contacts ICMJE: Contact: Seth Noar, PhD (919) 962-4075 noar@unc.edu

Listed Location Countries ICMJE: United States

Primary Outcome Measures:

Title	Time Frame	Definition
**PRIMARY OUTCOME**		
Mean susceptibility to vaping score	Week 3	The 3-item susceptibility to vaping scale assesses the extent to which adolescents are open to vaping. Response options are on a 1 to 4 scale. Overall scale scores are determined by summing scores of the 3 items and dividing by 3 (range 1-4), with higher scores

		representing a higher amount of the construct.

Secondary Outcome Measures:

Title	Time Frame	Definition
**SECONDARY OUTCOMES**		
Mean attention score	Week 2	The 1-item mean attention measure assesses the extent to which a participant reports that an ad grabs their attention. Response options are on a 1 to 5 scale, with higher scores representing a higher amount of the construct.
Mean negative affect score	Week 2	The 3-item negative affect scale assesses the extent to which an ad elicits negative emotion from the participant, such as fear or disgust. Response options are on a 1 to 5 scale. Overall scale scores are determined by summing scores of the 3 items and dividing by 3 (range 1-5), with higher scores representing a higher amount of the construct.
Mean cognitive elaboration score	Week 3	The 3-item mean cognitive elaboration scale assesses the extent to which a participant thought about the addictiveness or harmfulness of vaping in the past 7 days. Response options are on a 1 to 5 scale. Overall scale scores are determined by summing the scores of the 3 items and dividing by 3 (range 1-5), with higher scores representing a higher amount of the construct.
Mean social interactions score	Week 3	The 3-item mean social interactions scale assesses the extent to which a participant talked about the addictiveness or harmfulness of vaping with others in the past 7 days. Response options range on a scale from 1 (0 times) to 6 (11 or more times). Overall scale scores are determined by summing the scores (number of times) from the 3 items and dividing by 3 (range 0-33), with higher scores representing a higher amount of the construct.
Mean vaping health harm risk beliefs score	Week 3	The 3-item vaping health harm risk beliefs scale assesses the extent to which a participant believes that vaping will lead to health harms. Response options are on a 1 to 5 scale. Overall scales scores are determined by summing the scores of the 3 items and dividing by 3 (range 1-5), with higher scores representing a higher amount of the construct.
Mean vaping addiction risk beliefs score	Week 3	The 3-item vaping addiction risk beliefs scale assesses the extent to which a participant believes that vaping will lead to addiction. Response options are on a 1 to 5 scale. Overall scale scores are determined by summing the scores of the 3 items and dividing by 3 (range 1-5), with higher scores representing a higher amount of the construct.
Mean vaping attitudes score	Week 3	The 3-item vaping attitudes scale assesses a participant's attitude towards vaping (e.g., good/bad). Response options are on a 1 to 5 scale. Overall scale scores are determined by summing the scores of the 3 items and dividing by 3 (range 1-5), with higher scores representing a higher amount of the construct.
Proportion of participants who vape (e-cigarette use)	Week 3	Participants will be asked the number of days they vaped over the past 21 days. Vaping will be determined as follows: 0 days = non-vaper; 1 or more days = vaper

Mean smoking health harm risk belief score	Week 3	The 1-item mean smoking health harm risk belief measure assesses the extent to which a participant believes that smoking will lead to health harms. Response options are on a 1 to 5 scale, with higher scores representing a higher amount of the construct.
Mean smoking addiction risk belief score	Week 3	The 1-item mean smoking addiction risk belief measure assesses the extent to which a participant believes that smoking will lead to addiction. Response options are on a 1 to 5 scale, with higher scores representing a higher amount of the construct.
Mean smoking attitude score	Week 3	The 1-item mean smoking attitude measure assesses a participant's attitude toward smoking (i.e., good/bad). Response options are on a 1 to 5 scale, with higher scores representing a higher amount of the construct.
Mean susceptibility to smoking cigarettes score	Week 3	The 3-items mean susceptibility to smoking cigarettes scale assesses a participant's susceptibility to smoking cigarettes. Response options are on a 1 to 5 scale. Overall scale scores are determined by summing the scores of the 3 items and dividing by 3 (range 1-5), with higher scores representing a higher amount of the construct.
Proportion of participants who smoke cigarettes	Week 3	Participants will be asked the number of days they smoked cigarettes over the past 21 days. Cigarette smoking will be determined as follows: 0 days = non-smoker; 1 or more days = smoker

Other Outcome Measures:

**Title**	**Time Frame**	**Definition**
**OTHER OUTCOMES**		
Mean perceived message effectiveness score (UNC youth scale)	Week 2	The 3-item UNC youth mean perceived message effectiveness scale assesses a participant's judgement of an ad's impact on their vaping beliefs and behavior. Response options are on a 1 to 5 scale. Overall scores are determined by summing the scores of the 3 items and dividing by 3 (range 1-5), with higher scores representing a higher amount of the construct.
Mean perceived message effectiveness score (FDA Scale)	Week 2	The 6-item FDA mean perceived message effectiveness scale assesses a participant's general impressions of an ad. Response options are on a 1 to 5 scale. Overall scale scores are determined by summing the scores of the 6 items and dividing by 6 (range 1-5), with higher scores representing a higher amount of the construct.
Mean avoidance score	Week 2	The 3-item avoidance scale assesses the extent to which a participant wants to avoid processing an ad. Response options are on a 1 to 5 scale. Overall scores are determined by summing the scores of the 3 items and dividing by 3 (range 1-5), with higher scores representing a higher amount of the construct.
Mean Reactance scores	Week 2	The 1-item reactance measure assesses the extent to which an ad makes a participant feel annoyed. Response options are on a 1 to 5 scale, with higher scores representing a higher amount of the construct.
Mean Vaping social enhancement beliefs score	Week 3	The 3-item mean vaping social enhancement beliefs scale assesses what a participant believes about the social benefits of vaping (e.g., fit in better with friends). Response options are on a 1 to 5 scale. Overall scores are determined by summing the scores of the 3 items and dividing by 3 (range 1-5), with higher scores representing a

		higher amount of the construct.
Mean vaping affect regulation beliefs score	Week 3	The 3-item mean vaping affect regulation beliefs scale assesses what a participant believes about the experience of vaping (e.g., feel good). Response options are on a 1 to 5 scale. Overall scores are determined by summing the scores of the 3 items and dividing by 3 (range 1-5), with higher scores representing a higher amount of the construct.
Mean vaping injunctive norms score	Week 3	The 3-item mean vaping injunctive norms scale assesses a participant's perception of others' approval or disapproval of their vaping behavior. Response options are on a 1 to 5 scale. Overall scores are determined by summing the scores of the 3 items and dividing by 3 (range 1-5), with higher scores representing a higher amount of the construct.
Mean vaping refusal self-efficacy score	Week 3	The 3-item mean vaping refusal self-efficacy scale assesses a participant's confidence that they could refuse to vape in a social situation. Response options are on a 1 to 5 scale. Overall scores are determined by summing the scores of the 3 items and dividing by 3 (range 1-5), with higher scores representing a higher amount of the construct.

Note- no outcomes designated as safety issue

Locations

University of North Carolina, Chapel Hill
Chapel Hill, North Carolina, United States, 27599

Sponsors and Collaborators

UNC Lineberger Comprehensive Cancer Center
National Institutes of Health (NIH)
Food and Drug Administration (FDA)
National Cancer Institute (NCI)

Investigators

Principal Investigator: Seth Noar, PhD University of North Carolina, Chapel Hill

More Information

Responsible Party:	UNC Lineberger Comprehensive Cancer Center
ClinicalTrials.gov Identifier:	NCT04836455
Other Study ID Numbers:	19-3174<br>1R01CA246600-01 (U.S. NIH Grant/Contract)
Study First Received:	April 8, 2021
Last Updated:	February 9, 2022
**Health Authority:**	United States: Federal Government<br>United States: Institutional Review Board

Keywords provided by University of North Carolina, Chapel Hill:

Vaping prevention

Health communication
Tobacco use

Hypotheses and Analytic Plan

Hypotheses

The goal of this study is to determine whether exposure to vaping prevention advertisements reduce susceptibility to vaping. The **primary hypothesis** is that participants who view *The Real Cost* vaping prevention advertisements will have lower susceptibility to vaping compared to those who view neutral vaping control ads at 3 week follow-up.

The investigators also aim to examine the impact of *The Real Cost* vaping prevention ads on a series of other vaping-related outcomes. A**dditional hypotheses** are that, compared to exposure to neutral vaping ads, participants exposed to *The Real Cost* vaping prevention ads will report the following at 3-week follow-up:

- Lower susceptibility to vaping
- Higher health harm risk beliefs about vaping
- Higher addiction risk beliefs about vaping
- More negative attitudes toward vaping
- Less vaping
- Higher attention (assessed only at 2-week follow-up)
- Higher negative affect (assessed only at 2-week follow-up)
- Higher cognitive elaboration
- More social interactions

The investigators will also explore the effects of *The Real Cost* vaping prevention ads on cigarette smoking-related outcomes, compared to exposure to neutral vaping ads. There are **not have directional hypotheses** for the following outcomes:

- Susceptibility to smoking cigarettes
- Health harm risk beliefs about smoking cigarettes
- Addiction risk beliefs about smoking cigarettes
- Attitudes towards smoking cigarettes
- Cigarette smoking behavior

Finally, the investigators will explore whether health harms vaping prevention ads out-perform addiction *The Real Cost* ads. We have labeled this an exploratory activity because the study is powered to compare these ads to control ads but not to one another.

Primary analyses

The investigators will use a critical alpha of 0.05 in all statistical tests. Analyses of the primary and secondary outcomes will include all randomized participants according to the trial arm they were randomized to receive (i.e., intent-to-treat). The investigators will use multiple imputation to handle any missing data on predictors and full information maximum likelihood to handle any missing data on outcomes.

The investigators will report univariate and bivariate descriptive statistics for the primary and secondary outcomes.

For significance testing of the primary hypotheses, the investigators will compare those in *The Real Cost* trial arms (combined) to the control arm. The investigators will use latent curve models so that the investigators can

model change in repeated measures over time. After establishing a linear growth trajectory for each latent construct with the intercept coded to be at the last time point, the investigators will regress the latent intercept and slope factors for each outcome on an indicator of random assignment to *The Real Cost* trial arms or to the control arm. A significant effect on the intercept will indicate a main effect of treatment assignment on the mean level of each outcome at the last time point. A significant effect on the slope, if positive, will indicate that repeated viewing of the ads led to an increase in the difference across trial arms. If negative, it will indicate that the treatment effect diminished over time. As a secondary analysis, the investigators will examine whether there were significant differences in growth factor means as a function of assignment to the *The Real Cost* ad trial arms.

The investigators will also conduct the above analyses comparing those in the *The Real Cost* health harms trial arm to those in the *The Real Cost* addiction trial arm to explore whether there are differences across those trial arms.

The investigators will use a separate model for each of the study outcomes. To account for multiple comparisons, the investigators will use a Benjamini-Hochberg correction to maintain a 5% false detection rate.

Finally, the investigators will conduct exploratory moderation analyses on the primary outcome to examine whether the following variables moderate the impact of *The Real Cost* trial arms (compared to the control arm) on susceptibility to vaping: gender, age, race, Hispanic ethnicity, sexual orientation, parent's education, current vaper (vs. non-current vaper), and tobacco user in the household.

Sample size and power

The investigators plan to enroll 500 individuals per trial arm with the expectation that as many as 33% of participants will drop out of the study before week 3 follow-up. The investigators will use a FIML estimator so the investigators can retain all available data, including observed repeated measures from participants who go on to drop out of the study. With an estimated ICC=.7 (and thus a design effect of 2.4), the investigators have statistical power to detect an effect as small as Cohen's $d = .25$. This effect size is around the same magnitude as the effects observed with pilot data. The investigators expect that the effects will be larger than what is observed in pilot data because the latent variable approach will result in higher reliability measures.

Interim analysis

No interim analyses are planned.
