# Supplementary material for: Impact of Vaping Prevention Advertisements on US Adolescents: A Randomized Clinical Trial
Source: JAMA Netw Open. 2022 Oct 13;5(10):e2236370. doi: 10.1001/jamanetworkopen.2022.36370 (PMC9561946; doi:10.1001/jamanetworkopen.2022.36370)
Supplement: Supplement 2. — eTable 1. Vaping Prevention Video Advertisements Used in the Trial eFigure. Trial Design eTable 2. Primary and Secondary Outcome Measures Used in the Trial eTable 3. RCT Survey Codebook for Visits 1-4 eReferences. eAppendix. Additional Information on Statistical Models eTable 4. Participant Characteristics With Real Cost Groups Combined eTable 5. Item Completion at Visits 2, 3, and 4 [file jamanetwopen-e2236370-s002.pdf]

Supplemental Online Content

Noar SM, Gottfredson NC, Kieu T, et al. Impact of vaping prevention advertisements on US adolescents: a randomized clinical trial. *JAMA Netw Open*. 2022;5(10):e2236370. doi:10.1001/jamanetworkopen.2022.36370

eTable 1. Vaping Prevention Video Advertisements Used in the Trial

eFigure. Trial Design

eTable 2. Primary and Secondary Outcome Measures Used in the Trial

eTable 3. RCT Survey Codebook for Visits 1-4

eReferences.

eAppendix. Additional Information on Statistical Models

eTable 4. Participant Characteristics With Real Cost Groups Combined

eTable 5. Item Completion at Visits 2, 3, and 4

This supplemental material has been provided by the authors to give readers additional information about their work.

© 2022 Noar SM et al. *JAMA Network Open.*

eTable 1. Vaping Prevention Video Advertisements Used in the Trial

Condition	Ads	Prior recall n(%)
Real Cost – **Health Harms**	Epidemic: https://vimeo.com/325055700<br>Facts of Vaping: https://vimeo.com/413710792<br>Toxic Metals: https://vimeo.com/413711298	198 (39.3%)<br>189 (37.5%)<br>185 (36.7%)
Real Cost – **Addiction**	Hacked: https://vimeo.com/325055712<br>Vaping Mistake – Danny: https://vimeo.com/413711020<br>Nicotine addiction isn't pretty – Bathroom: https://vimeo.com/520033399	191 (37.7%)<br>186 (36.8%)<br>187 (37%)
**Control**	Definition: https://vimeo.com/514315064<br>Farming: https://vimeo.com/514315483<br>Manufacturing: https://vimeo.com/514315497	n/a

Note. Prior recall refers to reporting in the Visit 1 survey that participants had seen one or more of the ads in their assigned trial group previously.

© 2022 Noar SM et al. *JAMA Network Open.*

eFigure. Trial Design

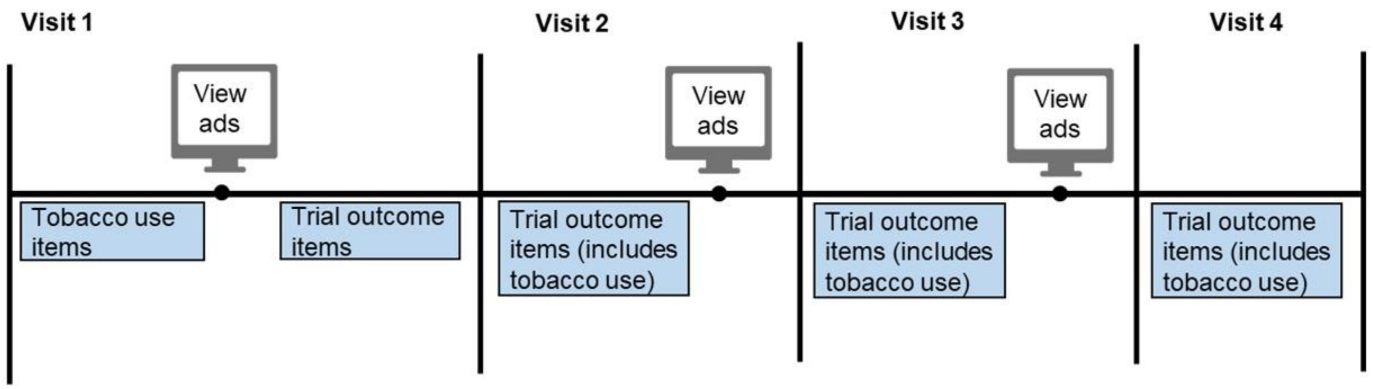

© 2022 Noar SM et al. *JAMA Network Open.*

eTable 2. Primary and Secondary Outcome Measures Used in the Trial

Construct, measure(s)	Response	Reference
PRIMARY OUTCOME		
Susceptibility to vaping		
Do you think you might use an e-cigarette or vape soon?	"Definitely not" (1)	
Do you think you will use an e-cigarette or vape in the next year?	...	Pierce et al., 1996
If one of your best friends were to offer you an e-cigarette or vape, would you use it?	"Definitely yes" (4)	
SECONDARY OUTCOMES		
Attention	"Not at all" (1)	Nonnemaker et al., 2015
How much do these ads grab your attention?	… "A great deal" (5)	
Negative affect	"Not at all" (1)	
How much do these ads make you feel scared?	…	Nonnemaker et al., 2015
How much do these ads make you feel disgusted?	"A great deal" (5)	
How much do these ads make you feel anxious?		
Cognitive elaboration	"Not at all" (1)	
In the past 7 days, how much did you think about the health harms of vaping?	…	Brewer et al., 2016
In the past 7 days, how much did you think about the addictiveness of vaping?	"A great deal" (5)	
In the past 7 days, how much did you think about reasons for not vaping?		
Social interactions	"0 times" (1)	
In the past 7 days, how many times did you talk to others about the health harms of vaping?	"1 time" (2)	
In the past 7 days, how many times did you talk to others about the addictiveness of vaping?	"2 times" (3)	Hall et al., 2015
In the past 7 days, how many times did you talk to others about reasons for not vaping?	"3 – 5 times" (4) "6 – 10 times" (5) "11 or more times" (6)	
Health harm risk beliefs	"Very unlikely" (1)	
How likely or unlikely is it that if you vape, you will damage your body?	…	Brennan et al., 2017
How likely or unlikely is it that if you vape, you will damage your lungs?	"Very likely" (5)	
How likely or unlikely is it that if you vape, you will harm your brain?		
Addiction risk beliefs	"Very unlikely" (1)	
How likely or unlikely is it that if you vape, you will become addicted to vaping?	…	Brennan et al., 2017
How likely or unlikely is it that if you vape, you will be controlled by vaping?	"Very likely" (5)	
How likely or unlikely is it that if you vape, you will be unable to stop vaping when you want to?		
Vaping attitudes		
Do you think vaping is…?	"Very bad" (1) … "Very good" (5)	Zhao et al., 2019
Do you think vaping is…?	"Very unenjoyable" (1) … "Very enjoyable" (5)	
Do you think vaping is…?	"Very harmful" (1) …	

© 2022 Noar SM et al. *JAMA Network Open.*

	"Very safe" (5)	
Days vaped<br>In the past 7 days, on how many days did you use an e-cigarette or vape?	0 – 7	Hyland et al., 2017
Health harm risk belief (smoking)	"Very unlikely" (1)<br>…<br>"Very likely" (5)	Brennan et al., 2017
How likely or unlikely is it that if you smoke cigarettes, you will damage your body?		
Addiction risk belief (smoking)	"Very unlikely" (1)<br>…<br>"Very likely" (5)	Brennan et al., 2017
How likely or unlikely is it that if you smoke cigarettes, you will become addicted to smoking?		
Smoking attitude		Zhao et al., 2019
Do you think smoking cigarettes is…?	"Very bad" (1)<br>…<br>"Very good" (5)	
Susceptibility to smoking cigarettes	"Definitely not" (1)<br>…<br>"Definitely yes" (4)	Pierce et al., 1996
Do you think you might smoke a cigarette soon?		
Do you think you will smoke a cigarette in the next year?		
If one of your best friends were to offer you a cigarette, would you smoke it?		
Days smoked<br>In the past 7 days, on how many days did you smoke a cigarette?	0 – 7	Hyland et al., 2017

© 2022 Noar SM et al. *JAMA Network Open.*

eTable 3. RCT Survey Codebook for Visits 1-4

Variable	Item	Response scale	Notes	Source	1	2	3	4
**A. TOBACCO USE**								
A10<br>Prompt	**The next questions are about e-cigarette use and vaping. The e-liquids used in these devices come in different flavors and nicotine concentrations.**<br><br>**People sometimes call these devices vapes, vape-pens, mods, pods, JUUL, Puff Bar, and by other names. The pictures below show examples of e-cigarettes and vapes.**<br><br>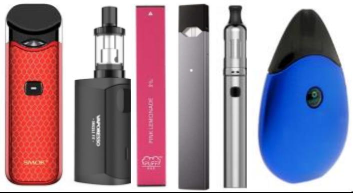		Intro language adapted from our past studies and the PATH youth extended questionnaire		X	X	X	X
A20<br>E-cigarette use – ever	Have you ever tried an e-cigarette, also called vaping, even one or two times?	1. No (skip to A60)<br>2. Yes	Skip pattern	Adapted from PATH (Hyland et al., 2017)	X			
A30<br>E- use – 30 days	In the past 30 days, on how many days did you use an e-cigarette or vape? [only ask if A20=2]	0-30 scrolling box (If 0, skip to A60)	Skip pattern	Adapted from PATH (Hyland et al., 2017)	X			
A40<br>E-cigarette use – 7 days	In the past 7 days, on how many days did you use an e-cigarette or vape? [T0: only ask if A30 >0]	0-7 scrolling box		Adapted from PATH (Hyland et al., 2017)	X	X	X	X
A60<br>Prompt	**The next questions are about smoking cigarettes and using other tobacco products.**				X	X	X	X

© 2022 Noar SM et al. *JAMA Network Open.*

Variable	Item	Response scale	Notes	Source	1	2	3	4
A70<br>Cigarette smoking – ever	Have you ever tried cigarette smoking, even one or two puffs?	1. No (skip to A110)<br>2. Yes	Skip pattern	Adapted from PATH (Hyland et al., 2017)	X			
A80<br>Cigarette use – 30 days	In the past 30 days, on how many days did you smoke a cigarette?<br>[only ask if A70=2]	0-30 scrolling box<br>(If 0, skip to A110)	Skip pattern	Adapted from PATH (Hyland et al., 2017)	X			
A90<br>Cigarette smoking – 7 days	In the past 7 days, on how many days did you smoke a cigarette?<br>[T0: only ask if A80 >0]	0-7 scrolling box<br>(Week 0: If 0, skip to A110)<br>(Week 1-3: If 0, skip to A130)		Adapted from PATH (Hyland et al., 2017)	X	X	X	X
A110<br>OTP use – ever	Have you ever used any of these products? Select all that apply.	1. Traditional cigars<br>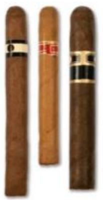<br>2. Cigarillos, filtered cigars or little cigars<br>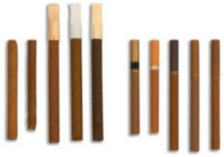<br>3. Pipe filled with tobacco<br>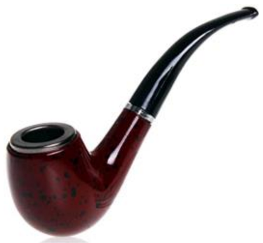<br>4. Hookah	T0: If None of the above is selected, skip to B10	Adapted from PATH (Hyland et al., 2017)	X			

© 2022 Noar SM et al. *JAMA Network Open.*

Variable	Item	Response scale	Notes	Source	1	2	3	4
		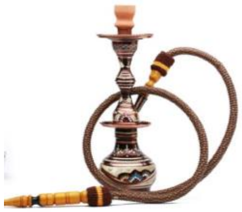<br>5. Smokeless tobacco (such as snus, moist snuff, dip, spit and chew)<br>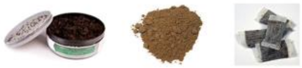<br>6. None of the above						
A120<br>OTP use – 30 days	Which of these tobacco products have you used in the <u>past 30 days?</u> Select all that apply.	1. Traditional cigars<br>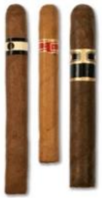<br>2. Cigarillos, filtered cigars or little cigars<br>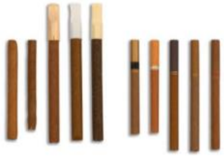<br>3. Pipe filled with tobacco<br>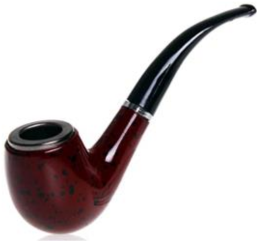<br>4. Hookah		Adapted from PATH (Hyland et al., 2017)	X			

© 2022 Noar SM et al. *JAMA Network Open.*

Variable	Item	Response scale	Notes	Source	1	2	3	4
		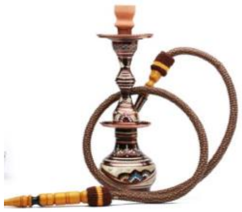<br>5. Smokeless tobacco (such as snus, moist snuff, dip, spit and chew)<br>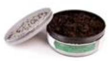 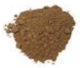 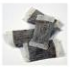<br>6. None of the above						
A130<br>OTP use – 7 days	Which of these tobacco products have you used in the <u>past 7 days?</u> Select all that apply.	1. Traditional cigars<br>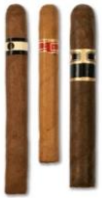<br>2. Cigarillos, filtered cigars or little cigars<br>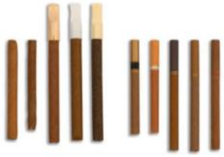<br>3. Pipe filled with tobacco<br>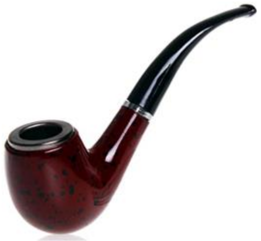<br>4. Hookah		Adapted from PATH (Hyland et al., 2017)	X	X	X	X

© 2022 Noar SM et al. *JAMA Network Open.*

Variable	Item	Response scale	Notes	Source	1	2	3	4
		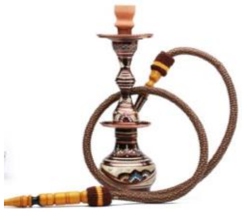<br>5. Smokeless tobacco (such as snus, moist snuff, dip, spit and chew)<br>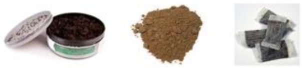<br>6. None of the above						
**B. VAPING AD EXPOSURE**<br>(Randomize participant to The Real Cost- Health Harms, The Real Cost- Addiction, or the Control ad condition)								
**First vaping ad exposure**								
B10<br>Prompt – first ad	**Now you are going to see three very short videos, one at a time. We will refer to these videos as ads.**<br><br>**On the next page, click the play button to watch the ad.**		Insert randomly selected ad; program 'next button' function to appear after 35 seconds		X	X	X	
B20<br>Recall	Before today, how many times had you seen this ad?	1. Not at all<br>2. Once<br>3. 2-4 times<br>4. 5-10 times<br>5. 11 or more times		Adapted from Southwell, Barmada, Hornik, and Maklan (2002)	X			
B30<br>Effects PME prompt	How much does this ad…		Matrix (B30)		X	X	X	
B30_1<br>Effects PME – worry	Make you worry about what vaping will do to you?	1=Not at all<br>2=Very little<br>3=Somewhat<br>4=Quite a bit<br>5=A great deal		Noar et al. (2021)	X	X	X	
B30_2<br>Effects PME – bad idea	Make you think vaping is a bad idea?	1=Not at all<br>2=Very little		Noar et al. (2021)	X	X	X	

© 2022 Noar SM et al. *JAMA Network Open.*

Variable	Item	Response scale	Notes	Source	1	2	3	4
		3=Somewhat<br>4=Quite a bit<br>5=A great deal						
B30_3<br>Effects PME – discourage	Discourage you from vaping?	1=Not at all<br>2=Very little<br>3=Somewhat<br>4=Quite a bit<br>5=A great deal		Noar et al. (2021)	X	X	X	
B40<br>Prompt	**Say whether you agree or disagree with the next statements.**				X	X	X	
B50<br>Message PME prompt	This ad…		Matrix (B50)		X	X	X	
B50_1<br>Message PME – attention	Grabs my attention.	1. Strongly disagree<br>2. Somewhat disagree<br>3. Neither agree nor disagree<br>4. Somewhat agree<br>5. Strongly agree		Davis, Nonnemaker, Duke, and Farrelly (2013)	X	X	X	
B50_2<br>Message PME – inform	Is informative.	1. Strongly disagree<br>2. Somewhat disagree<br>3. Neither agree nor disagree<br>4. Somewhat agree<br>5. Strongly agree		Davis et al. (2013)	X	X	X	
B50_3<br>Message PME – meaning	Is meaningful.	1. Strongly disagree<br>2. Somewhat disagree<br>3. Neither agree nor disagree<br>4. Somewhat agree<br>5. Strongly agree		Davis et al. (2013)	X	X	X	
B50_4<br>Message PME – remember	Is worth remembering.	1. Strongly disagree<br>2. Somewhat disagree<br>3. Neither agree nor disagree<br>4. Somewhat agree<br>5. Strongly agree		Davis et al. (2013)	X	X	X	
B50_5<br>Message PME – convince	Is convincing.	1. Strongly disagree<br>2. Somewhat disagree<br>3. Neither agree nor disagree<br>4. Somewhat agree<br>5. Strongly agree		Davis et al. (2013)	X	X	X	
B50_6<br>Message PME – powerful	Is powerful.	1. Strongly disagree<br>2. Somewhat disagree		Davis et al. (2013)	X	X	X	

© 2022 Noar SM et al. *JAMA Network Open.*

Variable	Item	Response scale	Notes	Source	1	2	3	4
		3. Neither agree nor disagree<br>4. Somewhat agree<br>5. Strongly agree						
**Second vaping ad exposure**								
B60<br>Prompt – second ad	**Now you are going to see another ad.**<br><br>**On the next page, click the play button to watch the ad.**		Insert randomly selected ad; program 'next button' function to appear after 35 seconds		X	X	X	
B70<br>Recall	Before today, how many times had you seen this ad?	1. Not at all<br>2. Once<br>3. 2-4 times<br>4. 5-10 times<br>5. 11 or more times		Adapted from Southwell et al. (2002)	X			
B80<br>Effects PME prompt	How much does this ad…		Matrix (B80)		X	X	X	
B80_1<br>Effects PME – worry	Make you worry about what vaping will do to you?	1=Not at all<br>2=Very little<br>3=Somewhat<br>4=Quite a bit<br>5=A great deal		Noar et al. (2021)	X	X	X	
B80_2<br>Effects PME – bad Idea	Make you think vaping is a bad idea?	1=Not at all<br>2=Very little<br>3=Somewhat<br>4=Quite a bit<br>5=A great deal		Noar et al. (2021)	X	X	X	
B80_3<br>Effects PME – discourage	Discourage you from vaping?	1=Not at all<br>2=Very little<br>3=Somewhat<br>4=Quite a bit<br>5=A great deal		Noar et al. (2021)	X	X	X	
B90<br>Prompt	**Say whether you agree or disagree with the next statements.**				X	X	X	
B100<br>Message PME prompt	This ad…		Matrix (B100)		X	X	X	
B100_1<br>Message PME – attention	Grabs my attention.	1. Strongly disagree<br>2. Somewhat disagree<br>3. Neither agree nor disagree<br>4. Somewhat agree<br>5. Strongly agree		Davis et al. (2013)	X	X	X	

© 2022 Noar SM et al. *JAMA Network Open.*

Variable	Item	Response scale	Notes	Source	1	2	3	4
B100_2<br>Message PME – inform	Is informative.	1. Strongly disagree<br>2. Somewhat disagree<br>3. Neither agree nor disagree<br>4. Somewhat agree<br>5. Strongly agree		Davis et al. (2013)	X	X	X	
B100_3<br>Message PME – meaning	Is meaningful.	1. Strongly disagree<br>2. Somewhat disagree<br>3. Neither agree nor disagree<br>4. Somewhat agree<br>5. Strongly agree		Davis et al. (2013)	X	X	X	
B100_4<br>Message PME – remember	Is worth remembering.	1. Strongly disagree<br>2. Somewhat disagree<br>3. Neither agree nor disagree<br>4. Somewhat agree<br>5. Strongly agree		Davis et al. (2013)	X	X	X	
B100_5<br>Message PME – convince	Is convincing.	1. Strongly disagree<br>2. Somewhat disagree<br>3. Neither agree nor disagree<br>4. Somewhat agree<br>5. Strongly agree		Davis et al. (2013)	X	X	X	
B100_6<br>Message PME – powerful	Is powerful.	1. Strongly disagree<br>2. Somewhat disagree<br>3. Neither agree nor disagree<br>4. Somewhat agree<br>5. Strongly agree		Davis et al. (2013)	X	X	X	
**Third vaping ad exposure**								
B110<br>Prompt – third ad	**Now you are going to see the last ad.**<br><br>**On the next page, click the play button to watch the ad.**		Insert randomly selected ad; program 'next button' function to appear after 35 seconds		X	X	X	
B120<br>Recall	Before today, how many times had you seen this ad?	1. Not at all<br>2. Once<br>3. 2-4 times<br>4. 5-10 times<br>5. 11 or more times		Adapted from Southwell et al. (2002)	X			
B130<br>Effects PME prompt	How much does this ad…		Matrix (B130)		X	X	X	

© 2022 Noar SM et al. *JAMA Network Open.*

Variable	Item	Response scale	Notes	Source	1	2	3	4
B130_1<br>Effects PME – worry	Make you worry about what vaping will do to you?	1=Not at all<br>2=Very little<br>3=Somewhat<br>4=Quite a bit<br>5=A great deal		Noar et al. (2021)	X	X	X	
B130_2<br>Effects PME – bad Idea	Make you think vaping is a bad idea?	1=Not at all<br>2=Very little<br>3=Somewhat<br>4=Quite a bit<br>5=A great deal		Noar et al. (2021)	X	X	X	
B130_3<br>Effects PME – discourage	Discourage you from vaping?	1=Not at all<br>2=Very little<br>3=Somewhat<br>4=Quite a bit<br>5=A great deal		Noar et al. (2021)	X	X	X	
B140<br>Prompt	**Say whether you agree or disagree with the next statements.**				X	X	X	
B150<br>Message PME prompt	This ad…		Matrix (B150)		X	X	X	
B150_1<br>Message PME – attention	Grabs my attention.	1. Strongly disagree<br>2. Somewhat disagree<br>3. Neither agree nor disagree<br>4. Somewhat agree<br>5. Strongly agree		Davis et al. (2013)	X	X	X	
B150_2<br>Message PME – inform	Is informative.	1. Strongly disagree<br>2. Somewhat disagree<br>3. Neither agree nor disagree<br>4. Somewhat agree<br>5. Strongly agree		Davis et al. (2013)	X	X	X	
B150_3<br>Message PME – meaning	Is meaningful.	1. Strongly disagree<br>2. Somewhat disagree<br>3. Neither agree nor disagree<br>4. Somewhat agree<br>5. Strongly agree		Davis et al. (2013)	X	X	X	
B150_4<br>Message PME – remember	Is worth remembering.	1. Strongly disagree<br>2. Somewhat disagree<br>3. Neither agree nor disagree<br>4. Somewhat agree<br>5. Strongly agree		Davis et al. (2013)	X	X	X	

© 2022 Noar SM et al. *JAMA Network Open.*

Variable	Item	Response scale	Notes	Source	1	2	3	4
B150_5<br>Message PME – convince	Is convincing.	1. Strongly disagree<br>2. Somewhat disagree<br>3. Neither agree nor disagree<br>4. Somewhat agree<br>5. Strongly agree		Davis et al. (2013)	X	X	X	
B150_6<br>Message PME – powerful	Is powerful.	1. Strongly disagree<br>2. Somewhat disagree<br>3. Neither agree nor disagree<br>4. Somewhat agree<br>5. Strongly agree		Davis et al. (2013)	X	X	X	
**C. VAPING MESSAGE REACTIONS**								
C10<br>Prompt	**Now, think about all 3 ads together when answering the next questions.**				X	X	X	
C20<br>Attention	How much do these ads grab your attention?	1=Not at all<br>2=Very little<br>3=Somewhat<br>4=Quite a bit<br>5=A great deal		Adapted from Nonnemaker, Choiniere, Farrelly, Kamyab, and Davis (2015)	X	X	X	
C30<br>Reactance	How much do these ads annoy you?	1=Not at all<br>2=Very little<br>3=Somewhat<br>4=Quite a bit<br>5=A great deal		Hall et al. (2017)	X	X	X	
C40<br>Negative affect prompt	How much do these ads make you feel…		Matrix (C40)<br>RANDOMIZE		X	X	X	
C40_1<br>Negative affect – scared	Scared?	1=Not at all<br>2=Very little<br>3=Somewhat<br>4=Quite a bit<br>5=A great deal		Adapted from Nonnemaker et al. (2015)	X	X	X	

© 2022 Noar SM et al. *JAMA Network Open.*

Variable	Item	Response scale	Notes	Source	1	2	3	4
C40_2<br>Negative affect – disgusted	Disgusted?	1=Not at all<br>2=Very little<br>3=Somewhat<br>4=Quite a bit<br>5=A great deal		Adapted from Nonnemaker et al. (2015)	X	X	X	
C40_3<br>Negative affect – anxious	Anxious?	1=Not at all<br>2=Very little<br>3=Somewhat<br>4=Quite a bit<br>5=A great deal		Adapted from Nonnemaker et al. (2015)	X	X	X	
C50<br>Avoidance prompt	How much do these ads make you want to…		Matrix (C50)<br>RANDOMIZE		X	X	X	
C50_1<br>Avoidance – look away	Look away?	1=Not at all<br>2=Very little<br>3=Somewhat<br>4=Quite a bit<br>5=A great deal		Adapted from PATH (Hyland et al., 2017)	X	X	X	
C50_2<br>Avoidance – what is said	Avoid thinking about what is being said?	1=Not at all<br>2=Very little<br>3=Somewhat<br>4=Quite a bit<br>5=A great deal		Adapted from PATH (Hyland et al., 2017)	X	X	X	
C50_3<br>Avoidance – something else	Think about something else?	1=Not at all<br>2=Very little<br>3=Somewhat<br>4=Quite a bit<br>5=A great deal		Adapted from PATH (Hyland et al., 2017)	X	X	X	
**D. VAPING BELIEFS**								
D10<br>Vaping beliefs prompt	**These next questions are about e-cigarette use and vaping.**				X	X	X	X
D20<br>Susceptibility to vaping – soon**	Do you think you might use an e-cigarette or vape soon?	1. Definitely not<br>2. Probably not<br>3. Probably yes<br>4. Definitely yes		Adapted from Pierce, Choi, Gilpin, Farkas, and Merritt (1996)	X	X	X	X

© 2022 Noar SM et al. *JAMA Network Open.*

Variable	Item	Response scale	Notes	Source	1	2	3	4
D30<br>Susceptibility to vaping – next year**	Do you think you will use an e-cigarette or vape in the next year?	1. Definitely not<br>2. Probably not<br>3. Probably yes<br>4. Definitely yes		Adapted from Pierce et al. (1996)	X	X	X	X
D40<br>Susceptibility to vaping – friend**	If one of your best friends were to offer you an e-cigarette or vape, would you use it?	1. Definitely not<br>2. Probably not<br>3. Probably yes<br>4. Definitely yes		Adapted from Pierce et al. (1996)	X	X	X	X
D50<br>Vaping attitudes – bad	Do you think vaping is…	1. Very bad<br>2. Somewhat bad<br>3. Neither good nor bad<br>4. Somewhat good<br>5. Very good		Adapted from Zhao, Roditis, and Alexander (2019)	X	X	X	X
D60<br>Vaping attitudes – unenjoyable	Do you think vaping is…	1. Very unenjoyable<br>2. Somewhat unenjoyable<br>3. Neither enjoyable nor unenjoyable<br>4. Somewhat enjoyable<br>5. Very enjoyable		Adapted from Zhao et al. (2019)	X	X	X	X
D70<br>Vaping attitudes – harmful	Do you think vaping is…	1. Very harmful<br>2. Somewhat harmful<br>3. Neither safe nor harmful<br>4. Somewhat safe<br>5. Very safe		Adapted from Zhao et al. (2019)	X	X	X	X
D80.<br>Health harm risk beliefs prompt	How likely or unlikely is it that if you vape, you will….		Matrix (D80)<br>RANDOMIZE		X	X	X	X
D80_1<br>Health harm risk beliefs – body	Damage your body?	1. Very unlikely<br>2. Somewhat unlikely<br>3. Neither likely nor unlikely<br>4. Somewhat likely<br>5. Very likely		Adapted from Brennan, Gibson, Kybert-Momjian, Liu, and Hornik (2017)	X	X	X	X
D80_2<br>Health harm risk beliefs – lungs	Damage your lungs?	1. Very unlikely<br>2. Somewhat unlikely<br>3. Neither likely nor unlikely<br>4. Somewhat likely<br>5. Very likely		Adapted from Brennan et al. (2017)	X	X	X	X

© 2022 Noar SM et al. *JAMA Network Open.*

Variable	Item	Response scale	Notes	Source	1	2	3	4
D80_3<br>Health harm risk beliefs – brain	Harm your brain?	1. Very unlikely<br>2. Somewhat unlikely<br>3. Neither likely nor unlikely<br>4. Somewhat likely<br>5. Very likely		Adapted from Brennan et al. (2017)	X	X	X	X
D90<br>Addiction risk beliefs prompt	How likely or unlikely is it that if you vape, you will….		Matrix (D90)<br>RANDOMIZE		X	X	X	X
D90_1<br>Addiction risk beliefs – addiction	Become addicted to vaping?	1. Very unlikely<br>2. Somewhat unlikely<br>3. Neither likely nor unlikely<br>4. Somewhat likely<br>5. Very likely		Adapted from Brennan et al. (2017)	X	X	X	X
D90_2<br>Addiction risk beliefs – controlled	Be controlled by vaping?	1. Very unlikely<br>2. Somewhat unlikely<br>3. Neither likely nor unlikely<br>4. Somewhat likely<br>5. Very likely		Adapted from Brennan et al. (2017)	X	X	X	X
D90_3<br>Addiction risk beliefs – stop	Be unable to stop vaping when you want to?	1. Very unlikely<br>2. Somewhat unlikely<br>3. Neither likely nor unlikely<br>4. Somewhat likely<br>5. Very likely		Adapted from Brennan et al. (2017)	X	X	X	X
D100.<br>Affect regulation expectancies prompt	How likely or unlikely is it that if you vape, you will….		Matrix (D100)<br>RANDOMIZE		X	X	X	X
D100_1<br>Affect regulation expectancies – relaxed	Feel relaxed?	1. Very unlikely<br>2. Somewhat unlikely<br>3. Neither likely nor unlikely<br>4. Somewhat likely<br>5. Very likely		Adapted from Pokhrel, Little, Fagan, Muranaka, and Herzog (2014)	X	X	X	X
D100_2<br>Affect regulation expectancies – good	Feel good?	1. Very unlikely<br>2. Somewhat unlikely<br>3. Neither likely nor unlikely<br>4. Somewhat likely<br>5. Very likely		Adapted from Pokhrel et al. (2014)	X	X	X	X

© 2022 Noar SM et al. *JAMA Network Open.*

Variable	Item	Response scale	Notes	Source	1	2	3	4
D100_3<br>Affect regulation expectancies – less stressed	Feel less stressed?	1. Very unlikely<br>2. Somewhat unlikely<br>3. Neither likely nor unlikely<br>4. Somewhat likely<br>5. Very likely		Adapted from Pokhrel et al. (2014)	X	X	X	X
D110<br>Social enhancement expectancies prompt	How likely or unlikely is it that if you vape, you will….		Matrix (D110)<br>RANDOMIZE		X	X	X	X
D110_1<br>Social enhancement expectancies – attractive	Look more attractive?	1. Very unlikely<br>2. Somewhat unlikely<br>3. Neither likely nor unlikely<br>4. Somewhat likely<br>5. Very likely		Adapted from Pokhrel et al. (2014)	X	X	X	X
D110_2<br>Social enhancement expectancies – fit in	Fit in better with your friends?	1. Very unlikely<br>2. Somewhat unlikely<br>3. Neither likely nor unlikely<br>4. Somewhat likely<br>5. Very likely		Adapted from Pokhrel et al. (2014)	X	X	X	X
D110_3<br>Social enhancement expectancies – status	Increase your status?	1. Very unlikely<br>2. Somewhat unlikely<br>3. Neither likely nor unlikely<br>4. Somewhat likely<br>5. Very likely		Adapted from Pokhrel et al. (2014)	X	X	X	X
D120<br>Injunctive norms prompt	People who are important to me…		Matrix (D120)<br>RANDOMIZE		X	X	X	X
D120_1<br>Injunctive norms – should not	Think I should not vape.	1. Strongly disagree<br>2. Somewhat disagree<br>3. Neither agree nor disagree<br>4. Somewhat agree<br>5. Strongly agree		Adapted from Conner and Sparks (2002)	X	X	X	X
D120_2<br>Injunctive norms – disapprove	Would disapprove of my vaping.	1. Strongly disagree<br>2. Somewhat disagree<br>3. Neither agree nor disagree<br>4. Somewhat agree<br>5. Strongly agree		Adapted from Conner and Sparks (2002)	X	X	X	X

© 2022 Noar SM et al. *JAMA Network Open.*

Variable	Item	Response scale	Notes	Source	1	2	3	4
D120_3<br>Injunctive norms – avoid	Want me to avoid vaping.<br>f	1. Strongly disagree<br>2. Somewhat disagree<br>3. Neither agree nor disagree<br>4. Somewhat agree<br>5. Strongly agree		Adapted from Conner and Sparks (2002)	X	X	X	X
D130<br>Refusal self-efficacy prompt	How sure are you that, if you really wanted to, you could avoid using vapes if…		Matrix (D130)<br>RANDOMIZE		X	X	X	X
D130_1<br>Refusal self-efficacy – party	You were at a party, bar, or club?	1. Not at all sure<br>2. A little sure<br>3. Somewhat sure<br>4. Very sure<br>5. Extremely sure		Adapted from Navarro, Hoffman, Ganz, Guillory, and Crankshaw (2021)	X	X	X	X
D130_2<br>Refusal self-efficacy – people	You were in a place where most people are vaping?	1. Not at all sure<br>2. A little sure<br>3. Somewhat sure<br>4. Very sure<br>5. Extremely sure		Adapted from Navarro et al. (2021)	X	X	X	X
D130_3<br>Refusal self-efficacy – offer	Someone you know offers you a vape?	1. Not at all sure<br>2. A little sure<br>3. Somewhat sure<br>4. Very sure<br>5. Extremely sure		Adapted from Navarro et al. (2021)	X	X	X	X
D140<br>Seven days prompt	**For these next questions, think about the past 7 days.**				X	X	X	X
D150<br>Cognitive elaboration prompt	In the past 7 days, how much did you think about…		Matrix (D150)<br>RANDOMIZE		X	X	X	X
D150_1<br>Cognitive elaboration – health harms	The health harms of vaping?	1=Not at all<br>2=Very little<br>3=Somewhat<br>4=Quite a bit<br>5=A great deal		Adapted from Brewer et al. (2016)	X	X	X	X

© 2022 Noar SM et al. *JAMA Network Open.*

Variable	Item	Response scale	Notes	Source	1	2	3	4
D150_2<br>Cognitive elaboration – addictiveness	The addictiveness of vaping?	1=Not at all<br>2=Very little<br>3=Somewhat<br>4=Quite a bit<br>5=A great deal		Adapted from Brewer et al. (2016)	X	X	X	X
D150_3<br>Cognitive elaboration – reasons	Reasons for not vaping?	1=Not at all<br>2=Very little<br>3=Somewhat<br>4=Quite a bit<br>5=A great deal		Adapted from Brewer et al. (2016)	X	X	X	X
D160<br>Social interactions prompt	In the past 7 days, how many times did you talk to others about …		Matrix (D160)<br>RANDOMIZE		X	X	X	X
D160_1<br>Social interactions – health harms	The health harms of vaping?	1. 0 times<br>2. 1 time<br>3. 2 times<br>4. 3 – 5 times<br>5. 6 – 10 times<br>6. 11 or more times		Adapted from Hall et al. (2015)	X	X	X	X
D160_2<br>Social interactions – addictiveness	The addictiveness of vaping?	1. 0 times<br>2. 1 time<br>3. 2 times<br>4. 3 – 5 times<br>5. 6 – 10 times<br>6. 11 or more times		Adapted from Hall et al. (2015)	X	X	X	X
D160_3<br>Social interactions – reasons	Reasons for not vaping?	1. 0 times<br>2. 1 time<br>3. 2 times<br>4. 3 – 5 times<br>5. 6 – 10 times<br>6. 11 or more times		Adapted from Hall et al. (2015)	X	X	X	X
**E. SMOKING BELIEFS (NON-TARGETED BEHAVIOR)**								
E10<br>Smoking beliefs prompt	**These next questions are about cigarette smoking.**				X	X	X	X

© 2022 Noar SM et al. *JAMA Network Open.*

Variable	Item	Response scale	Notes	Source	1	2	3	4
E20<br>Susceptibility to smoking cigarettes – soon	Do you think you might smoke a cigarette soon?	1. Definitely not<br>2. Probably not<br>3. Probably yes<br>4. Definitely yes		Adapted from Pierce et al. (1996)	X	X	X	X
E30<br>Susceptibility to smoking cigarettes – next year	Do you think you will smoke a cigarette in the next year?	1. Definitely not<br>2. Probably not<br>3. Probably yes<br>4. Definitely yes		Adapted from Pierce et al. (1996)	X	X	X	X
E40<br>Susceptibility to smoking cigarettes – friend	If one of your best friends were to offer you a cigarette, would you smoke it?	1. Definitely not<br>2. Probably not<br>3. Probably yes<br>4. Definitely yes		Adapted from Pierce et al. (1996)	X	X	X	X
E50<br>Smoking attitudes	Do you think smoking cigarettes is…	1. Very bad<br>2. Somewhat bad<br>3. Neither good nor bad<br>4. Somewhat good<br>5. Very good		Adapted from Zhao et al. (2019)	X	X	X	X
E60<br>Smoking risk beliefs prompt	How likely or unlikely is it that if you smoke cigarettes, you will….		Matrix (E60)<br>RANDOMIZE		X	X	X	X
E60_1<br>Smoking risk beliefs – health harm	Damage your body.	1. Very unlikely<br>2. Somewhat unlikely<br>3. Neither likely nor unlikely<br>4. Somewhat likely<br>5. Very likely		Adapted from Brennan et al. (2017)	X	X	X	X
E60_2<br>Smoking risk beliefs – addiction	Become addicted to smoking.	1. Very unlikely<br>2. Somewhat unlikely<br>3. Neither likely nor unlikely<br>4. Somewhat likely<br>5. Very likely		Adapted from Brennan et al. (2017)	X	X	X	X
**F. SMOKING AD EXPOSURE (AIM 3 ONLY)**<br>(Randomize participant to The Real Cost- Health Harms, The Real Cost- Addiction, or the Control smoking ad condition)								
**First smoking ad exposure**								
F10<br>Prompt – first ad	**Now you are going to see three very short videos, one at a time. We will refer to these videos as ads.**		Insert randomly selected ad; program 'next button' function to					X

© 2022 Noar SM et al. *JAMA Network Open.*

Variable	Item	Response scale	Notes	Source	1	2	3	4
	**On the next page, click the play button to watch the ad.**		appear after 35 seconds					
F20<br>Smoking ad recall	Before today, how many times have you seen this ad?	1. Not at all<br>2. Once<br>3. 2-4 times<br>4. 5-10 times<br>5. 11 or more times		Adapted from Southwell et al. (2002)				X
F30<br>Effects PME prompt	How much does this ad…		Matrix (F30)					X
F30_1<br>Effects PME – worry	Make you worry about what smoking cigarettes will do to you?	1=Not at all<br>2=Very little<br>3=Somewhat<br>4=Quite a bit<br>5=A great deal		Noar et al. (2021)				X
F30_2<br>Effects PME – bad idea	Make you think smoking cigarettes is a bad idea?	1=Not at all<br>2=Very little<br>3=Somewhat<br>4=Quite a bit<br>5=A great deal		Noar et al. (2021)				X
F30_3<br>Effects PME – discourage	Discourage you from smoking cigarettes?	1=Not at all<br>2=Very little<br>3=Somewhat<br>4=Quite a bit<br>5=A great deal		Noar et al. (2021)				X
F40<br>Prompt	**Say whether you agree or disagree with the next statements.**							X
F50<br>Message PME prompt	This ad…		Matrix (F50)					X
F50_1<br>Message PME – attention	Grabs my attention.	1. Strongly disagree<br>2. Somewhat disagree<br>3. Neither agree nor disagree<br>4. Somewhat agree<br>5. Strongly agree		Davis et al. (2013)				X
F50_2<br>Message PME – inform	Is informative.	1. Strongly disagree<br>2. Somewhat disagree<br>3. Neither agree nor disagree		Davis et al. (2013)				X

© 2022 Noar SM et al. *JAMA Network Open.*

Variable	Item	Response scale	Notes	Source	1	2	3	4
		4. Somewhat agree<br>5. Strongly agree						
F50_3<br>Message PME – meaning	Is meaningful.	1. Strongly disagree<br>2. Somewhat disagree<br>3. Neither agree nor disagree<br>4. Somewhat agree<br>5. Strongly agree		Davis et al. (2013)				X
F50_4<br>Message PME – remember	Is worth remembering.	1. Strongly disagree<br>2. Somewhat disagree<br>3. Neither agree nor disagree<br>4. Somewhat agree<br>5. Strongly agree		Davis et al. (2013)				X
F50_5<br>Message PME – convince	Is convincing.	1. Strongly disagree<br>2. Somewhat disagree<br>3. Neither agree nor disagree<br>4. Somewhat agree<br>5. Strongly agree		Davis et al. (2013)				X
F50_6<br>Message PME – powerful	Is powerful.	1. Strongly disagree<br>2. Somewhat disagree<br>3. Neither agree nor disagree<br>4. Somewhat agree<br>5. Strongly agree		Davis et al. (2013)				X
**Second smoking ad exposure**								
F60<br>Prompt – second ad	**Now you are going to see another ad.**<br><br>**On the next page, click the play button to watch the ad.**		Insert randomly selected ad; program 'next button' function to appear after 35 seconds					X
F70<br>Smoking ad recall	Before today, how many times have you seen this ad?	1. Not at all<br>2. Once<br>3. 2-4 times<br>4. 5-10 times<br>5. 11 or more times		Adapted from Southwell et al. (2002)				X
F80<br>Effects PME prompt	How much does this ad…		Matrix (F80)					X
F80_1<br>Effects PME – worry	Make you worry about what smoking cigarettes will do to you?	1=Not at all<br>2=Very little<br>3=Somewhat		Noar et al. (2021)				X

© 2022 Noar SM et al. *JAMA Network Open.*

Variable	Item	Response scale	Notes	Source	1	2	3	4
		4=Quite a bit<br>5=A great deal						
F80_2<br>Effects PME – bad idea	Make you think smoking cigarettes is a bad idea?	1=Not at all<br>2=Very little<br>3=Somewhat<br>4=Quite a bit<br>5=A great deal		Noar et al. (2021)				X
F80_3<br>Effects PME – discourage	Discourage you from smoking cigarettes?	1=Not at all<br>2=Very little<br>3=Somewhat<br>4=Quite a bit<br>5=A great deal		Noar et al. (2021)				X
F90<br>Prompt	**Say whether you agree or disagree with the next statements.**							X
F100<br>Message PME prompt	This ad…		Matrix (F100)					X
F100_1<br>Message PME – attention	Grabs my attention.	1. Strongly disagree<br>2. Somewhat disagree<br>3. Neither agree nor disagree<br>4. Somewhat agree<br>5. Strongly agree		Davis et al. (2013)				X
F100_2<br>Message PME – inform	Is informative.	1. Strongly disagree<br>2. Somewhat disagree<br>3. Neither agree nor disagree<br>4. Somewhat agree<br>5. Strongly agree		Davis et al. (2013)				X
F100_3<br>Message PME – meaning	Is meaningful.	1. Strongly disagree<br>2. Somewhat disagree<br>3. Neither agree nor disagree<br>4. Somewhat agree<br>5. Strongly agree		Davis et al. (2013)				X
F100_4<br>Message PME – remember	Is worth remembering.	1. Strongly disagree<br>2. Somewhat disagree<br>3. Neither agree nor disagree<br>4. Somewhat agree<br>5. Strongly agree		Davis et al. (2013)				X
F100_5<br>Message PME – convince	Is convincing.	1. Strongly disagree<br>2. Somewhat disagree		Davis et al. (2013)				X

© 2022 Noar SM et al. *JAMA Network Open.*

Variable	Item	Response scale	Notes	Source	1	2	3	4
		3. Neither agree nor disagree<br>4. Somewhat agree<br>5. Strongly agree						
F100_6<br>Message PME – powerful	Is powerful.	1. Strongly disagree<br>2. Somewhat disagree<br>3. Neither agree nor disagree<br>4. Somewhat agree<br>5. Strongly agree		Davis et al. (2013)				X
**Third smoking ad exposure**								
F110<br>Prompt – third ad	**Now you are going to see the last ad.**<br><br>**On the next page, click the play button to watch the ad.**		Insert randomly selected ad; program 'next button' function to appear after 35 seconds					X
F120<br>Smoking ad recall	Before today, how many times have you seen this ad?	1. Not at all<br>2. Once<br>3. 2-4 times<br>4. 5-10 times<br>5. 11 or more times		Adapted from Southwell et al. (2002)				X
F130<br>Effects PME prompt	How much does this ad…		Matrix (F130)					X
F130_1<br>Effects PME – worry	Make you worry about what smoking cigarettes will do to you?	1=Not at all<br>2=Very little<br>3=Somewhat<br>4=Quite a bit<br>5=A great deal		Noar et al. (2021)				X
F130_2<br>Effects PME – bad idea	Make you think smoking cigarettes is a bad idea?	1=Not at all<br>2=Very little<br>3=Somewhat<br>4=Quite a bit<br>5=A great deal		Noar et al. (2021)				X
F130_3<br>Effects PME – discourage	Discourage you from smoking cigarettes?	1=Not at all<br>2=Very little<br>3=Somewhat<br>4=Quite a bit<br>5=A great deal		Noar et al. (2021)				X
F140<br>Prompt	**Say whether you agree or disagree with the next statements.**							X

© 2022 Noar SM et al. *JAMA Network Open.*

Variable	Item	Response scale	Notes	Source	1	2	3	4
F150<br>Message PME prompt	This ad…		Matrix (F150)					X
F150_1<br>Message PME – attention	Grabs my attention.	1. Strongly disagree<br>2. Somewhat disagree<br>3. Neither agree nor disagree<br>4. Somewhat agree<br>5. Strongly agree		Davis et al. (2013)				X
F150_2<br>Message PME – inform	Is informative.	1. Strongly disagree<br>2. Somewhat disagree<br>3. Neither agree nor disagree<br>4. Somewhat agree<br>5. Strongly agree		Davis et al. (2013)				X
F150_3<br>Message PME – meaning	Is meaningful.	1. Strongly disagree<br>2. Somewhat disagree<br>3. Neither agree nor disagree<br>4. Somewhat agree<br>5. Strongly agree		Davis et al. (2013)				X
F150_4<br>Message PME – remember	Is worth remembering.	1. Strongly disagree<br>2. Somewhat disagree<br>3. Neither agree nor disagree<br>4. Somewhat agree<br>5. Strongly agree		Davis et al. (2013)				X
F150_5<br>Message PME – convince	Is convincing.	1. Strongly disagree<br>2. Somewhat disagree<br>3. Neither agree nor disagree<br>4. Somewhat agree<br>5. Strongly agree		Davis et al. (2013)				X
F150_6<br>Message PME – powerful	Is powerful.	1. Strongly disagree<br>2. Somewhat disagree<br>3. Neither agree nor disagree<br>4. Somewhat agree<br>5. Strongly agree		Davis et al. (2013)				X
**G. SMOKING MESSAGE REACTIONS (AIM 3 ONLY)**								

© 2022 Noar SM et al. *JAMA Network Open.*

Variable	Item	Response scale	Notes	Source	1	2	3	4
G10<br>Prompt	**Now, think about all 3 ads together when answering the following questions.**							X
G20<br>Attention	How much do these ads grab your attention?	1=Not at all<br>2=Very little<br>3=Somewhat<br>4=Quite a bit<br>5=A great deal		Adapted from Nonnemaker et al. (2015)				X
G30<br>Reactance	How much do these ads annoy you?	1=Not at all<br>2=Very little<br>3=Somewhat<br>4=Quite a bit<br>5=A great deal		Hall et al. (2017)				X
G40<br>Negative affect	How much do these ads make you feel…		Matrix (G50)<br>RANDOMIZE					X
G40_1<br>Negative affect – scared	Scared?	1=Not at all<br>2=Very little<br>3=Somewhat<br>4=Quite a bit<br>5=A great deal		Adapted from Nonnemaker et al. (2015)				X
G40_2<br>Negative affect – disgusted	Disgusted?	1=Not at all<br>2=Very little<br>3=Somewhat<br>4=Quite a bit<br>5=A great deal		Adapted from Nonnemaker et al. (2015)				X
G40_3<br>Negative affect – anxious	Anxious?	1=Not at all<br>2=Very little<br>3=Somewhat<br>4=Quite a bit<br>5=A great deal		Adapted from Nonnemaker et al. (2015)				X
G50<br>Avoidance	How much do these ads make you want to…		Matrix (G60)<br>RANDOMIZE					X
G50_1<br>Avoidance – look away	Look away?	1=Not at all<br>2=Very little<br>3=Somewhat<br>4=Quite a bit<br>5=A great deal		Adapted from PATH (Hyland et al., 2017)				X

© 2022 Noar SM et al. *JAMA Network Open.*

Variable	Item	Response scale	Notes	Source	1	2	3	4
G50_2<br>Avoidance – avoiding thinking	Avoid thinking about what is being said?	1=Not at all<br>2=Very little<br>3=Somewhat<br>4=Quite a bit<br>5=A great deal		Adapted from PATH (Hyland et al., 2017)				X
G50_3<br>Avoidance – think something else	Think about something else?	1=Not at all<br>2=Very little<br>3=Somewhat<br>4=Quite a bit<br>5=A great deal		Adapted from PATH (Hyland et al., 2017)				X
G60<br>Cognitive elaboration	How much do these ads cause you to think about…		Matrix (G70)<br>RANDOMIZE					X
G60_1<br>Cognitive elaboration – health harms	The health harms of smoking cigarettes?	1=Not at all<br>2=Very little<br>3=Somewhat<br>4=Quite a bit<br>5=A great deal		Adapted from Brewer et al. (2016)				X
G60_2<br>Cognitive elaboration - addictiveness	The addictiveness of smoking cigarettes?	1=Not at all<br>2=Very little<br>3=Somewhat<br>4=Quite a bit<br>5=A great deal		Adapted from Brewer et al. (2016)				X
G60_3<br>Cognitive elaboration – reasons	Reasons for not smoking cigarettes?	1=Not at all<br>2=Very little<br>3=Somewhat<br>4=Quite a bit<br>5=A great deal		Adapted from Brewer et al. (2016)				X
**H. SMOKING BELIEFS (AIM 3 ONLY)**								
H10<br>Smoking beliefs prompt	**These next questions are about cigarette smoking.**							X
H20<br>Susceptibility to smoking cigarettes – soon	Do you think you might smoke a cigarette soon?	1. Definitely not<br>2. Probably not<br>3. Probably yes<br>4. Definitely yes		Adapted from Pierce et al. (1996)				X

© 2022 Noar SM et al. *JAMA Network Open.*

Variable	Item	Response scale	Notes	Source	1	2	3	4
H30<br>Susceptibility to smoking cigarettes – next year	Do you think you will smoke a cigarette in the next year?	1. Definitely not<br>2. Probably not<br>3. Probably yes<br>4. Definitely yes		Adapted from Pierce et al. (1996)				X
H40<br>Susceptibility to smoking cigarettes – friend	If one of your best friends were to offer you a cigarette, would you smoke it?	1. Definitely not<br>2. Probably not<br>3. Probably yes<br>4. Definitely yes		Adapted from Pierce et al. (1996)				X
H50<br>Smoking attitudes – bad	Do you think smoking cigarettes is…	1. Very bad<br>2. Somewhat bad<br>3. Neither good nor bad<br>4. Somewhat good<br>5. Very good		Adapted from Zhao et al. (2019)				X
H60<br>Smoking attitudes – unenjoyable	Do you think smoking cigarettes is…	1. Very unenjoyable<br>2. Somewhat unenjoyable<br>3. Neither enjoyable nor unenjoyable<br>4. Somewhat enjoyable<br>5. Very enjoyable		Adapted from Zhao et al. (2019)				X
H70<br>Smoking attitudes – Harmful	Do you think smoking cigarettes is…	1. Very harmful<br>2. Somewhat harmful<br>3. Neither safe nor harmful<br>4. Somewhat safe<br>5. Very safe		Adapted from Zhao et al. (2019)				X
H80<br>Smoking health harm risk beliefs prompt	How likely or unlikely is it that if you smoke cigarettes, you will….		Matrix (H70)<br>RANDOMIZE					X
H80_1<br>Smoking health harm risk beliefs – body	Damage your body	1. Very unlikely<br>2. Somewhat unlikely<br>3. Neither likely nor unlikely<br>4. Somewhat likely<br>5. Very likely		Adapted from Brennan et al. (2017)				X
H80_2<br>Smoking health harm risk beliefs – lungs	Damage your lungs	1. Very unlikely<br>2. Somewhat unlikely<br>3. Neither likely nor unlikely<br>4. Somewhat likely<br>5. Very likely		Adapted from Brennan et al. (2017)				X
H80_3<br>Smoking health harm risk beliefs – brain	Harm your brain	1. Very unlikely<br>2. Somewhat unlikely<br>3. Neither likely nor unlikely<br>4. Somewhat likely<br>5. Very likely		Adapted from Brennan et al. (2017)				X

© 2022 Noar SM et al. *JAMA Network Open.*

Variable	Item	Response scale	Notes	Source	1	2	3	4
H90<br>Smoking addiction risk beliefs prompt	How likely or unlikely is it that if you smoke cigarettes, you will….		Matrix (H80)<br>RANDOMIZE					X
H90_1<br>Smoking addiction risk beliefs – addiction	Become addicted to smoking	1. Very unlikely<br>2. Somewhat unlikely<br>3. Neither likely nor unlikely<br>4. Somewhat likely<br>5. Very likely		Adapted from Brennan et al. (2017)				X
H90_2<br>Smoking addiction risk beliefs – control	Be controlled by smoking	1. Very unlikely<br>2. Somewhat unlikely<br>3. Neither likely nor unlikely<br>4. Somewhat likely<br>5. Very likely		Adapted from Brennan et al. (2017)				X
H90_3<br>Smoking addiction risk beliefs – stop	Be unable to stop smoking when you want to	1. Very unlikely<br>2. Somewhat unlikely<br>3. Neither likely nor unlikely<br>4. Somewhat likely<br>5. Very likely		Adapted from Brennan et al. (2017)				X
H100<br>Seven days prompt	**For these next questions, think about the next 7 days.**							X
H110<br>Social interactions prompt	In the next 7 days, how many times do you think you will talk to others about…							X
H110_1<br>Social interactions – health harms	The health harms of smoking cigarettes?	1. 0 times<br>2. 1 time<br>3. 2 times<br>4. 3 – 5 times<br>5. 6 – 10 times<br>6. 11 or more times	Matrix (G110)<br>RANDOMIZE	Adapted from Hall et al. (2015)				X
H110_2<br>Social interactions – addictiveness	The addictiveness of smoking cigarettes?	1. 0 times<br>2. 1 time<br>3. 2 times<br>4. 3 – 5 times<br>5. 6 – 10 times<br>6. 11 or more times		Adapted from Hall et al. (2015)				X

© 2022 Noar SM et al. *JAMA Network Open.*

Variable	Item	Response scale	Notes	Source	1	2	3	4
H110_3<br>Social interactions – reasons	Reasons for not smoking cigarettes?	1. 0 times<br>2. 1 time<br>3. 2 times<br>4. 3 – 5 times<br>5. 6 – 10 times<br>6. 11 or more times		Adapted from Hall et al. (2015)				X
**I. MARIJUANA**								
I10<br>Marijuana prompt	**Next are some questions about marijuana, which can be smoked, vaped, or eaten. By marijuana we also mean pot, weed, or cannabis.**				X			X
I20<br>Marijuana ever	Have you ever used marijuana, even one or two times?	1. No<br>2. Yes		Adapted from YRBSS (Centers for Disease Control and Prevention, 2015)	X			
I30<br>Marijuana past 30 days	[only ask if I20=2]<br>In the past 30 days, on how many days did you use marijuana?	0-30 scrolling box<br>(If 0, skip to J10)	Skip pattern	Adapted from YRBSS (Centers for Disease Control and Prevention, 2015)	X			
I40<br>Past 30 number of days prompt	[only ask if I30>0]<br>In the past 30 days, when you used marijuana, on how many days did you…		Matrix (I40)		X			
I40_1<br>Past 30 number of days – vape marijuana	Vape marijuana (for example, using a vape pen, mod, pod, or JUUL)?	0-30 scrolling box		Developed for this study.	X			
I40_2<br>Past 30 number of days – smoke marijuana	Smoke marijuana (for example, using a joint, bowl, pipe, bong)?	0-30 scrolling box		Developed for this study.	X			
I40_3<br>Past 30 number of days – eat marijuana	Eat food that contained marijuana?	0-30 scrolling box		Developed for this study.	X			
I50<br>Marijuana past 3 weeks	In the past 21 days (about 3 weeks), on how many days did you use marijuana?	0-21 scrolling box<br>(If 0, skip to L10)	Skip pattern	Adapted from YRBSS (Centers for Disease				X

© 2022 Noar SM et al. *JAMA Network Open.*

Variable	Item	Response scale	Notes	Source	1	2	3	4
				Control and Prevention, 2015)				
I60<br>Past 3 weeks number of days prompt	[only ask if I30>0]<br>In the past 21 days, when you used marijuana, on how many days did you…		Matrix (I60)					X
I60_1<br>Past 3 weeks number of days – vape marijuana	Vape marijuana (for example, using a vape pen, mod, pod, or JUUL)?	0-21 scrolling box		Developed for this study.				X
I60_2<br>Past 3 weeks number of days – smoke marijuana	Smoke marijuana (for example, using a joint, bowl, pipe, bong)?	0-21 scrolling box		Developed for this study.				X
I60_3<br>Past 3 weeks number of days – eat marijuana	Eat food that contained marijuana?	0-21 scrolling box		Developed for this study.				X
**J. ALCOHOL**								
J10<br>Alcohol prompt	**The following questions ask about interactions that you have had with doctors, nurses, or other healthcare providers.**				X			
J20<br>Alcohol ever	Has a doctor, nurse, or other healthcare provider ever spoken with you about drinking alcohol?	1. No<br>2. Yes<br>3. I don't remember	Skip pattern: If J10=2, then ask J20-J60, if J10=1 or 3, skip to end of block	Developed for this study.	X			
J30<br>Alcohol talk	The last time a healthcare provider talked with you about drinking alcohol, what did you talk about? Please select all that apply.	1. If I have tried drinking alcohol<br>2. The risks of drinking alcohol<br>3. Limiting how much I drink alcohol<br>4. Something else<br>5. I don't remember		Developed for this study.	X			
J40<br>Alcohol provider type	The last time a healthcare provider talked with you about drinking alcohol, who specifically did you talk to?	1. Doctor or physician's assistant<br>2. Nurse<br>3. Someone else<br>4. I don't know	Select all that apply	Developed for this study.	X			
J50<br>Alcohol context	The last time a healthcare provider talked with you about drinking alcohol, how did the conversation start?	1. The healthcare provider brought it up on their own		Developed for this study.	X			

© 2022 Noar SM et al. *JAMA Network Open.*

Variable	Item	Response scale	Notes	Source	1	2	3	4
		2. The healthcare provider brought it up because I reported that I had used alcohol<br>3. I brought it up<br>4. My parents asked about it<br>5. I don't remember						
J60<br>Alcohol parents	The last time a healthcare provider talked with you about drinking alcohol, who else was in the room?	1. Nobody else<br>2. My parents/guardians<br>3. Another family member<br>4. Someone else<br>5. I don't remember		Developed for this study.	X			
**K. DEMOGRAPHICS**								
K10<br>Demographics prompt	**We are asking the questions in this final section to better understand who completed our survey.**				X			
K20<br>Gender	Select the option that best describes your gender.	1=Female<br>2=Male<br>3=Transgender<br>4=Nonbinary or gender queer<br>5=Questioning<br>6=Prefer not to say		Adapted from the Williams Institute (Williams Institute, 2014)	X			
K30<br>Age	How old are you?	Scroll box, 13-17 years old		Developed for this study.	X			
K40<br>Hispanic ethnicity	Are you of Hispanic, Latino or Spanish origin?	1. No<br>2. Yes		Adapted from the 2020 U.S. Census	X			
K50<br>Race	What is your race? Select all that apply.	1. White<br>2. Black or African American<br>3. American Indian or Alaska Native<br>4. Asian<br>5. Native Hawaiian or other Pacific Islander<br>6. Some other race (please specify)	Select all that apply	Adapted from the 2020 U.S. Census	X			

© 2022 Noar SM et al. *JAMA Network Open.*

Variable	Item	Response scale	Notes	Source	1	2	3	4
K60<br>Sexual Orientation	This question is about your sexual orientation. Do you consider yourself to be...	1=Straight or heterosexual<br>2=Gay, lesbian, or bisexual<br>3=Prefer to self describe:__________<br>4=Prefer not to say		Adapted from the Williams Institute (Williams Institute, 2009)	X			
K70<br>Grade	What is the highest grade you have completed in school?	1= 5th Grade<br>2= 6th Grade<br>3= 7th Grade<br>4= 8th Grade<br>5= 9th Grade<br>6= 10th Grade<br>7= 11th Grade<br>8= 12th Grade<br>9= Graduated high school (or equivalent, such as GED)<br>10= In college<br>11= I have dropped out of school		—	X			
K80<br>Mother's Education	What is the highest degree or level of school <u>your mother or female guardian</u> has completed?	1. Less than high school<br>2. High school graduate (or equivalent, such as GED)<br>3. Some college<br>4. Associate degree<br>5. Bachelor's degree<br>6. Master's degree<br>7. Doctorate degree<br>8. I don't know		—	X			
K90<br>Father's Education	What is the highest degree or level of school <u>your father or male guardian</u> has completed?	1. Less than high school<br>2. High school graduate (or equivalent, such as GED)<br>3. Some college<br>4. Associate degree<br>5. Bachelor's degree<br>6. Master's degree<br>7. Doctorate degree<br>8. I don't know		—	X			

© 2022 Noar SM et al. *JAMA Network Open.*

Variable	Item	Response scale	Notes	Source	1	2	3	4
K100<br>Smoking in home	Does anyone who lives with you now...<br>(select all that apply)	1. Smoke cigarettes?<br>2. Smoke cigars, cigarillos, or little cigars?<br>3. Use chewing tobacco, snuff, or dip ?<br>4. Use e-cigarettes or vape?<br>5. Use another form of tobacco (e.g., hookah or waterpipe, snus) ?<br>6. No one who lives with me now uses any form of tobacco.	Select all that apply	Adapted from CDC's NYTS 2020 Questionnaire (Centers for Disease Control and Prevention, 2020)	X			
K110<br>Social Media	How often do you use the following social media?<br>1. Facebook<br>2. Instagram<br>3. Reddit<br>4. Snapchat<br>5. Twitter<br>6. TikTok<br>7. Tumblr<br>8. YouTube	1. Never<br>2. Almost never<br>3. At least once a week<br>4. Once a day<br>5. Several times a day<br>6. Almost constantly	Matrix<br><br>Randomize order of social media sites	Adapted from AP-NORC<br><br>(Associated Press-NORC Center, 2018)	X			
**L. FEASIBILITY & ACCEPTABILITY**								
L10<br>Survey feedback	Is there anything you would like to tell us about this survey?<br><br>Please tell us below:	[open-ended response]		Developed for this study.	X	X	X	
L20<br>Prompt	**Please answer these final questions about being in this study.**							X
L30<br>Length of study	For this study, we asked you to take one survey each week for four weeks. Would you say that four weeks was…	1. Too short<br>2. About right<br>3. Too long		Developed for this study.				X
L40<br>Length of study open-ended	Please tell us more about this below:	[open-ended response]		Developed for this study.				X

© 2022 Noar SM et al. *JAMA Network Open.*

Variable	Item	Response scale	Notes	Source	1	2	3	4
L50<br>Length of surveys	Would you say the length of the surveys you took each week was…	1. Too short<br>2. About right<br>3. Too long		Developed for this study.				X
L60<br>Length of surveys open-ended	Please tell us more about this below:	[open-ended response]		Developed for this study.				X
L70<br>Survey difficulty	How easy or hard was it to take the surveys?	1. Very easy<br>2. Somewhat easy<br>3. Neither easy nor hard<br>4. Somewhat hard<br>5. Very hard		Developed for this study.				X
L80<br>Survey difficulty open-ended	Please tell us more about this below:	[open-ended response]		Developed for this study.				X
L90<br>Video amount	You watched three short videos in each survey. Would you say that three videos per survey was…	1. Too few<br>2. About right<br>3. Too many		Developed for this study.				X
L100<br>Video amount open-ended	Please tell us more about this below:	[open-ended response]		Developed for this study.				X
L110<br>Incentive	You will receive incentives (for example, gift cards or points) for taking each survey. Would you say these incentives are…	1. Not enough<br>2. About right<br>3. Too much		Developed for this study.				X
L120<br>Incentive open-ended	Please tell us more about this below:	[open-ended response]		Developed for this study.				X
L130<br>Enroll again	If you had the chance, would you enroll in this study again?	1. Definitely wouldn’t<br>2. Probably wouldn’t<br>3. Not sure<br>4. Probably would<br>5. Definitely would		Developed for this study.				X
L140<br>Enroll again open-ended	Please tell us more about this below:	[open-ended response]		Developed for this study.				X
L150<br>Changes to study	If we do this study again, what should we change? Please tell us below:	[open-ended response]		Developed for this study.				
L160<br>Anything else	Is there anything else you would like to tell us about the surveys or about participating in this study? Please tell us below:	[open-ended response]		Developed for this study.				X

© 2022 Noar SM et al. *JAMA Network Open.*

Variable	Item	Response scale	Notes	Source	1	2	3	4
**END OF SURVEY PROMPTS**								
EOS prompt	**You have now finished this survey. Thank you for your participation.**<br><br>**Keep an eye out for your next survey, which will be sent to the email address you provided in about 1 week.**<br><br>**We look forward to hearing from you then!**				X	X	X	
EOS final survey debrief	**Thank you for completing this study. You have finished the final survey!**<br><br>**If you want to learn more about the risks of vaping and smoking, you can view "The Real Cost" website that was developed by FDA's Center for Tobacco Products.**<br><br>**https://therealcost.betobaccofree.hhs.gov/**							X

© 2022 Noar SM et al. *JAMA Network Open.*

eReferences

Associated Press-NORC Center. (2018). *The April 2018 AP-NORC Center Poll*. Retrieved from: https://apnorc.org/wp-content/uploads/2020/02/April-2018-AP-NORC-Poll-Topline_Complete-Final.pdf

Brennan, E., Gibson, L. A., Kybert-Momjian, A., Liu, J., & Hornik, R. C. (2017). Promising themes for antismoking campaigns targeting youth and young adults. *Tobacco Regulatory Science, 3*(1), 29-46. doi:10.18001/TRS.3.1.4

Brewer, N. T., Hall, M. G., Noar, S. M., Parada, H., Stein-Seroussi, A., Bach, L. E., . . . Ribisl, K. M. (2016). Effect of pictorial cigarette pack warnings on changes in smoking behavior: A randomized clinical trial. *JAMA Internal Medicine, 176*(7), 905-912. doi:10.1001/jamainternmed.2016.2621

Centers for Disease Control and Prevention. (2015). *Youth risk behavior surveillance system (YRBSS)*. Retrieved from Atlanta, GA:

Centers for Disease Control and Prevention. (2020). National Youth Tobacco Survey (2020) questionnaire. Retrieved from https://www.cdc.gov/tobacco/data_statistics/surveys/nyts/pdfs/2020/2020-NYTS-Questionnaire-508.pdf. https://www.cdc.gov/tobacco/data_statistics/surveys/nyts/pdfs/2020/2020-NYTS-Questionnaire-508.pdf

Conner, M., & Sparks, P. (2002). Theory of planned behaviour and health behaviour. In M. Conner & P. Norman (Eds.), *Predicting health behavior: Research and practice with social cognition models*. New York, NY: Open University Press.

Davis, K. C., Nonnemaker, J., Duke, J., & Farrelly, M. C. (2013). Perceived effectiveness of cessation advertisements: The importance of audience reactions and practical implications for media campaign planning. *Health Communication, 28*(5), 461-472. doi:10.1080/10410236.2012.696535

Hall, M. G., Peebles, K., Bach, L. E., Noar, S. M., Ribisl, K. M., & Brewer, N. T. (2015). Social interactions sparked by pictorial warnings on cigarette packs. *International Journal of Environmental Research and Public Health, 12*(10), 13195-13208. doi:10.3390/ijerph121013195

Hall, M. G., Sheeran, P., Noar, S. M., Ribisl, K. M., Boynton, M. H., & Brewer, N. T. (2017). A brief measure of reactance to health warnings. *Journal of Behavioral Medicine, 40*(3), 520-529. doi:10.1007/s10865-016-9821-z

Hyland, A., Ambrose, B. K., Conway, K. P., Borek, N., Lambert, E., Carusi, C., . . . Compton, W. M. (2017). Design and methods of the Population Assessment of Tobacco and Health (PATH) study. *Tobacco Control*, *26*(4), 371-378. doi:10.1136/tobaccocontrol-2016-052934

Navarro, M. A., Hoffman, L., Ganz, O., Guillory, J., & Crankshaw, E. C. (2021). Those who believe they can, do: The relationship between smoking avoidance beliefs, perceived risks of smoking, and behavior in a sexual and gender minority young adult sample. *Addictive Behaviors*, *113*. doi:10.1016/j.addbeh.2020.106733

Noar, S. M., Gottfredson, N. C., Vereen, R., Kurtzman, R., Mendel Sheldon, J., Adams, E. T., . . . Brewer, N. T. (2021). Development of the UNC perceived message effectiveness scale for youth. *Tobacco Control, doi: 10.1136/tobaccocontrol-2021-056929.*

Nonnemaker, J. M., Choiniere, C. J., Farrelly, M. C., Kamyab, K., & Davis, K. C. (2015). Reactions to graphic health warnings in the United States. *Health Education Research, 30*(1), 46-56. doi:10.1093/her/cyu036

Pierce, J. P., Choi, W. S., Gilpin, E. A., Farkas, A. J., & Merritt, R. K. (1996). Validation of susceptibility as a predictor of which adolescents take up smoking in the United States. *Health Psychology*, *15*(5), 355-361. doi:10.1037/0278-6133.15.5.355

Pokhrel, P., Little, M. A., Fagan, P., Muranaka, N., & Herzog, T. A. (2014). Electronic cigarette use outcome expectancies among college students. *Addictive Behaviors, 39*(6), 1062-1065. doi:10.1016/j.addbeh.2014.02.014

Southwell, B. G., Barmada, C. H., Hornik, R. C., & Maklan, D. M. (2002). Can we measure encoded exposure? Validation evidence from a national campaign. *Journal of Health Communication, 7*(5), 445-453. doi:10.1080/10810730290001800

© 2022 Noar SM et al. *JAMA Network Open.*

Williams Institute. (2009). *Best practices for asking about sexual orientation on surveys*. Retrieved from Los Angeles, CA: https://williamsinstitute.law.ucla.edu/publications/smart-so-survey/
Williams Institute. (2014). *Best practices for asking questions to identify transgender and other gender minority respondents on population-based surveys*. Retrieved from Los Angeles, CA: https://williamsinstitute.law.ucla.edu/publications/geniuss-trans-pop-based-survey/
Zhao, X., Roditis, M. L., & Alexander, T. N. (2019). Fear and Humor Appeals in "The Real Cost" Campaign: Evidence of Potential Effectiveness in Message Pretesting. *American Journal of Preventive Medicine, 56*(2s1), S31-S39. doi:10.1016/j.amepre.2018.07.033

© 2022 Noar SM et al. *JAMA Network Open.*

eAppendix. Additional Information on Statistical Models

Change in primary and secondary outcomes was not linear because at Visit 1 participants completed outcome measures following presentation of the ad but at later visits a week after last seeing an ad. We included effects for each visit (i.e., visit number was dummy coded and treated as a set of nominal predictors (parameters η_1 through η_3 in Equation 1). Visit indicators were also included as interactions with treatment arm (parameters η_5 through η_7 in Equation 1). The resulting model enabled us to report group differences for each visit without imposing linearity assumptions. In Equation 1, η_0 represents the expected value of the outcome at Visit 4 for the control group and η_4 represents the treatment effect at Visit 4. The parameter δ_i reflects individual differences in the mean of the outcome, y_{ti}, and r_{ti} is the time-varying residual term.

$$y_{ti} = \eta_0 + \eta_1 Visit1 + \eta_2 Visit2 + \eta_3 Visit3 + \eta_4 Tx_i + \eta_5 Visit1 * Tx_i + \eta_6 Visit2 * Tx_i + \eta_7 Visit3 * Tx_i + \delta_i + r_{ti} \quad (1)$$

We probed the interaction terms to identify the treatment effect at each visit and report these results.

Inter-individual variation in the intercept factor was statistically significant, reflecting individual differences in baseline mean levels of the outcomes. Variances for the other visit effects were fixed to zero due to nonsignificant inter-individual variation, reflecting a constant effect across individuals.

eTable 4. Participant Characteristics With Real Cost Groups Combined

	Overall (%)	Both *Real Cost* groups (n=1010)	Control group (*n*=504)
Age (*M*, SD)	15.22 (1.18)	15.20 (1.18)	15.28 (1.18)
Gender identity			
Male	1140 (75.3)	747 (74.0)	393 (78.0)
Female	358 (23.6)	248 (24.6)	110 (21.8)
Other responses	15 (1.1)	15 (1.5)	1 (0.2)
Race			
White	1081 (71.4)	712 (70.5)	369 (73.2)
Black or African-American	371 (24.5)	253 (25)	118 (23.4)
Asian	14 (0.9)	8 (0.8)	6 (1.2)
Native Hawaiian or other Pacific Islander	4 (0.3)	4 (0.4)	0 (0.0)
Multiracial	33 (2.2)	25 (2.5)	8 (1.6)
Other*	10 (<0.1)	7 (<0.1)	3 (<0.1)
Missing	1 (<0.1)	1 (<0.1)	0 (0.0)
Hispanic	176 (11.6)	115 (11.4)	61 (12.1)
Sexual orientation			
Heterosexual	1432 (94.6)	947 (93.8)	485 (96.2)
Lesbian, gay, bisexual, pansexual, or queer	61 (4.1)	48 (4.8)	13 (2.6)
Prefer not to say/Missing	20 (1.3)	15 (1.5)	6 (1.2)
Adolescent's education			
<High school	233 (15.4)	157 (15.5)	76 (15.1)
Some high school	1067 (70.6)	715 (70.8)	352 (69.8)
High school or GED	123 (8.1)	79 (7.8)	44 (8.7)
Some college	85 (5.6)	55 (5.4)	30 (6.0)
Dropped out of school	5 (0.3)	3 (0.3)	2 (0.4)
Missing	1 (0.1)	1 (0.1)	0 (0.0)
Mother's education			
<Bachelor's degree	262 (17.3)	187 (18.5)	75 (14.9)
Bachelor's degree	533 (35.2)	352 (34.9)	181 (35.9)
Master's degree	578 (38.2)	378 (37.4)	200 (39.7)
Doctorate degree	123 (8.1)	82 (8.1)	41 (8.1)
Missing	18 (1.2)	11 (1.1)	7 (1.4)
Father's education			
<Bachelor's degree	169 (11.1)	122 (12.1)	47 (8.8)
Bachelor's degree	353 (23.3)	235 (23.3)	118 (23.4)
Master's degree	699 (46.2)	463 (45.8)	236 (47.4)
Doctorate degree	273 (18.0)	176 (17.4)	97 (19.2)
Missing	20 (1.3)	14 (1.4)	6 (1.2)
Lived with somebody who…			
Smoked cigarettes	539 (35.6)	340 (33.7)	199 (39.5)
Used e-cigarettes or vapes	443 (29.3)	279 (27.6)	164 (32.5)
Used chewing tobacco, snuff, or dip	218 (14.4)	145 (14.4)	73 (14.5)
Smoked cigars, cigarillos, or little cigars	203 (13.4)	126 (12.5)	77 (15.3)
Used another form of tobacco	119 (7.9)	82 (8.1)	37 (7.3)
Tobacco use, in the past 30 days			
Used e-cigarette	993 (65.6)	660 (65.3)	333 (66.1)
Used cigarette	895 (59.1)	589 (58.3)	306 (60.7)
Used OTP	950 (62.7)	625 (61.9)	325 (64.5)

Note. SD = standard deviation. GED = general education diploma. OTP = other tobacco product use.
*Denotes participants self-reporting race as "other"

© 2022 Noar SM et al. *JAMA Network Open.*

eTable 5. Item Completion at Visits 2, 3, and 4

Visit 2 (1371 Initiated Visit 2)			Visit 3 (1346 Initiated Visit 3)			Visit 4 (1377 Initiated Visit 4)		
**Item Name**	**# of Participants Who Completed Item**	**% of Participants Who Completed Item**	**Item Name**	**# of Participants Who Completed Item**	**# of Participants Who Completed Item**	**Item Name**	**# of Participants Who Completed Item**	**% of Participants Who Completed Item**
T1_A40	1371	100.00%	T2_A40	1343	99.78%	T3_A40	1376	99.93%
T1_A90	1371	100.00%	T2_A90	1346	100.00%	T3_A90	1377	100.00%
T1_D20	1371	100.00%	T2_D20	1346	100.00%	T3_D20	1377	100.00%
T1_D30	1371	100.00%	T2_D30	1346	100.00%	T3_D30	1377	100.00%
T1_D40	1371	100.00%	T2_D40	1346	100.00%	T3_D40	1377	100.00%
T1_D50	1371	100.00%	T2_D50	1346	100.00%	T3_D50	1377	100.00%
T1_D60	1371	100.00%	T2_D60	1346	100.00%	T3_D60	1377	100.00%
T1_D70	1371	100.00%	T2_D70	1346	100.00%	T3_D70	1377	100.00%
T1_D80_1	1369	99.85%	T2_D80_1	1346	100.00%	T3_D80_1	1376	99.93%
T1_D80_2	1370	99.93%	T2_D80_2	1346	100.00%	T3_D80_2	1376	99.93%
T1_D80_3	1370	99.93%	T2_D80_3	1346	100.00%	T3_D80_3	1376	99.93%
T1_D90_1	1369	99.85%	T2_D90_1	1346	100.00%	T3_D90_1	1375	99.85%
T1_D90_2	1370	99.93%	T2_D90_2	1346	100.00%	T3_D90_2	1376	99.93%
T1_D90_3	1369	99.85%	T2_D90_3	1346	100.00%	T3_D90_3	1375	99.85%
T1_D100_1	1369	99.85%	T2_D100_1	1346	100.00%	T3_D100_1	1376	99.93%
T1_D100_2	1368	99.78%	T2_D100_2	1346	100.00%	T3_D100_2	1376	99.93%
T1_D100_3	1369	99.85%	T2_D100_3	1346	100.00%	T3_D100_3	1375	99.85%
T1_D110_1	1369	99.85%	T2_D110_1	1346	100.00%	T3_D110_1	1376	99.93%
T1_D110_2	1369	99.85%	T2_D110_2	1346	100.00%	T3_D110_2	1376	99.93%
T1_D110_3	1369	99.85%	T2_D110_3	1346	100.00%	T3_D110_3	1376	99.93%
T1_D120_1	1370	99.93%	T2_D120_1	1344	99.85%	T3_D120_1	1376	99.93%
T1_D120_2	1370	99.93%	T2_D120_2	1345	99.93%	T3_D120_2	1376	99.93%
T1_D120_3	1370	99.93%	T2_D120_3	1345	99.93%	T3_D120_3	1376	99.93%
T1_D130_1	1369	99.85%	T2_D130_1	1346	100.00%	T3_D130_1	1376	99.93%
T1_D130_2	1370	99.93%	T2_D130_2	1346	100.00%	T3_D130_2	1376	99.93%
T1_D130_3	1370	99.93%	T2_D130_3	1346	100.00%	T3_D130_3	1376	99.93%

© 2022 Noar SM et al. *JAMA Network Open.*

T1_D150_1	1370	99.93%	T2_D150_1	1345	99.93%	T3_D150_1	1375	99.85%
T1_D150_2	1370	99.93%	T2_D150_2	1346	100.00%	T3_D150_2	1375	99.85%
T1_D150_3	1370	99.93%	T2_D150_3	1346	100.00%	T3_D150_3	1376	99.93%
T1_D160_1	1370	99.93%	T2_D160_1	1346	100.00%	T3_D160_1	1376	99.93%
T1_D160_2	1369	99.85%	T2_D160_2	1346	100.00%	T3_D160_2	1375	99.85%
T1_D160_3	1370	99.93%	T2_D160_3	1346	100.00%	T3_D160_3	1376	99.93%
T1_E20	1370	99.93%	T2_E20	1345	99.93%	T3_E20	1376	99.93%
T1_E30	1370	99.93%	T2_E30	1345	99.93%	T3_E30	1376	99.93%
T1_E40	1370	99.93%	T2_E40	1344	99.85%	T3_E40	1376	99.93%
T1_E50	1367	99.71%	T2_E50	1344	99.85%	T3_E50	1370	99.49%
T1_E60_1	1370	99.93%	T2_E60_1	1346	100.00%	T3_E60_1	1373	99.71%
T1_E60_2	1370	99.93%	T2_E60_2	1346	100.00%	T3_E60_2	1371	99.56%
T1_B30_1	1370	99.93%	T2_B30_1	1345	99.93%	T3_F20	1375	99.85%
T1_B30_2	1370	99.93%	T2_B30_2	1345	99.93%	T3_F30_1	1375	99.85%
T1_B30_3	1370	99.93%	T2_B30_3	1345	99.93%	T3_F30_2	1375	99.85%
T1_B50_1	1370	99.93%	T2_B50_1	1345	99.93%	T3_F30_3	1375	99.85%
T1_B50_2	1370	99.93%	T2_B50_2	1345	99.93%	T3_F50_1	1375	99.85%
T1_B50_3	1370	99.93%	T2_B50_3	1345	99.93%	T3_F50_2	1375	99.85%
T1_B50_4	1370	99.93%	T2_B50_4	1345	99.93%	T3_F50_3	1375	99.85%
T1_B50_5	1370	99.93%	T2_B50_5	1345	99.93%	T3_F50_4	1375	99.85%
T1_B50_6	1370	99.93%	T2_B50_6	1345	99.93%	T3_F50_5	1375	99.85%
T1_B80_1	1370	99.93%	T2_B80_1	1345	99.93%	T3_F50_6	1375	99.85%
T1_B80_2	1370	99.93%	T2_B80_2	1345	99.93%	T3_F70	1375	99.85%
T1_B80_3	1370	99.93%	T2_B80_3	1345	99.93%	T3_F80_1	1375	99.85%
T1_B100_1	1370	99.93%	T2_B100_1	1345	99.93%	T3_F80_2	1375	99.85%
T1_B100_2	1370	99.93%	T2_B100_2	1345	99.93%	T3_F80_3	1375	99.85%
T1_B100_3	1370	99.93%	T2_B100_3	1345	99.93%	T3_F100_1	1375	99.85%
T1_B100_4	1370	99.93%	T2_B100_4	1345	99.93%	T3_F100_2	1375	99.85%
T1_B100_5	1370	99.93%	T2_B100_5	1345	99.93%	T3_F100_3	1375	99.85%
T1_B100_6	1370	99.93%	T2_B100_6	1345	99.93%	T3_F100_4	1375	99.85%
T1_B130_1	1370	99.93%	T2_B130_1	1345	99.93%	T3_F100_5	1375	99.85%
T1_B130_2	1370	99.93%	T2_B130_2	1345	99.93%	T3_F100_6	1375	99.85%

© 2022 Noar SM et al. *JAMA Network Open.*

T1_B130_3	1370	99.93%	T2_B130_3	1345	99.93%	T3_F120	1375	99.85%
T1_B150_1	1370	99.93%	T2_B150_1	1345	99.93%	T3_F130_1	1375	99.85%
T1_B150_2	1370	99.93%	T2_B150_2	1345	99.93%	T3_F130_2	1375	99.85%
T1_B150_3	1370	99.93%	T2_B150_3	1345	99.93%	T3_F130_3	1375	99.85%
T1_B150_4	1370	99.93%	T2_B150_4	1345	99.93%	T3_F150_1	1375	99.85%
T1_B150_5	1370	99.93%	T2_B150_5	1345	99.93%	T3_F150_2	1375	99.85%
T1_B150_6	1370	99.93%	T2_B150_6	1345	99.93%	T3_F150_3	1375	99.85%
T1_C20	1370	99.93%	T2_C20	1345	99.93%	T3_F150_4	1375	99.85%
T1_C30	1370	99.93%	T2_C30	1345	99.93%	T3_F150_5	1375	99.85%
T1_C40_1	1370	99.93%	T2_C40_1	1345	99.93%	T3_F150_6	1375	99.85%
T1_C40_2	1370	99.93%	T2_C40_2	1345	99.93%	T3_G20	1375	99.85%
T1_C40_3	1370	99.93%	T2_C40_3	1345	99.93%	T3_G30	1375	99.85%
T1_C50_1	1369	99.85%	T2_C50_1	1344	99.85%	T3_G40_1	1375	99.85%
T1_C50_2	1370	99.93%	T2_C50_2	1345	99.93%	T3_G40_2	1375	99.85%
T1_C50_3	1369	99.85%	T2_C50_3	1345	99.93%	T3_G40_3	1375	99.85%
						T3_G50_1	1374	99.78%
						T3_G50_2	1375	99.85%
						T3_G50_3	1374	99.78%
						T3_G60_1	1375	99.85%
						T3_G60_2	1375	99.85%
						T3_G60_3	1374	99.78%
						T3_H20	1374	99.78%
						T3_H30	1374	99.78%
						T3_H40	1375	99.85%
						T3_H50	1375	99.85%
						T3_H60	1375	99.85%
						T3_H70	1375	99.85%
						T3_H80_1	1375	99.85%
						T3_H80_2	1375	99.85%
						T3_H80_3	1375	99.85%
						T3_H90_1	1375	99.85%
						T3_H90_2	1375	99.85%

© 2022 Noar SM et al. *JAMA Network Open.*

						T3_H90_3	1375	99.85%
						T3_H110_1	1374	99.78%
						T3_H110_2	1375	99.85%
						T3_H110_3	1375	99.85%
						T3_I50	1375	99.85%

© 2022 Noar SM et al. *JAMA Network Open.*
