# Supplementary material for: Impact of Vaping Prevention Advertisements on US Adolescents: A Randomized Clinical Trial
Source: JAMA Netw Open. 2022 Oct 13;5(10):e2236370. doi: 10.1001/jamanetworkopen.2022.36370 (PMC9561946; doi:10.1001/jamanetworkopen.2022.36370)
Supplement: Supplement 3. — Data Sharing Statement [file jamanetwopen-e2236370-s003.pdf]

Data Sharing Statement

Noar. Impact of Vaping Prevention Advertisements on US Adolescents. *JAMA Netw Open*. Published October 13, 2022. doi:10.1001/jamanetworkopen.2022.36370

Data
Data available: No

Additional Information
Explanation for why data not available: The data will be made available at a later point after all manuscripts from the RCT have been published.
